# Associations between physical activity, sedentary behaviour and self-rated health among the general population of children and adolescents: a systematic review and meta-analysis

**DOI:** 10.1186/s12889-020-09447-1

**Published:** 2020-09-03

**Authors:** Tong Zhang, Guohua Lu, Xiu Yun Wu

**Affiliations:** School of Public Health, Weifang Medical University, 7166 Baotong West Street, Weifang, 261053 Shandong China

**Keywords:** Children, Adolescents, Physical activity, Sedentary behaviour, Self-rated health, Systematic review, Meta-analysis

## Abstract

**Background:**

Self-rated health (SRH) is an indicator that captures a person’s perception of their overall health status. The relationship between physical activity (PA), sedentary behaviour (SB) and SRH has been investigated in systematic reviews among adult and elderly populations. No systematic review to date has synthesized the relationship between PA, SB and SRH among children and adolescents. The purpose of this systematic review and meta-analysis was to synthesize the associations between PA, SB and SRH in the general population of children and adolescents and to investigate the dose-response relationship between PA, SB and SRH.

**Methods:**

We conducted a computer search for English language studies in the databases of MEDLINE, EMBASE and PSYCINFO that were published between 1946 and 2019. We searched PubMed, Google Scholar, and the references of the identified publications for additional studies. A meta-analysis was employed to synthesize the associations between PA, SB respectively and SRH. The dose-response association was tested using a random effects meta-regression model. The review was reported following the Preferred Reporting Items for Systematic reviews and Meta-Analyses (PRISMA) guidelines.

**Results:**

Sixty-eight published articles were included in the final review, including 59 cross-sectional and nine longitudinal studies. We found evidence that PA was associated with better SRH, and SB was associated with lower SRH among children and adolescents. A dose-response relationship between PA and SRH was observed, where a higher level of PA was associated with better SRH than a lower level of PA. The relationship between PA, SB and SRH was observed in both boys and girls, and did not show a significant gender difference.

**Conclusions:**

The findings in the systematic review suggest that health intervention programmes targeting promoting PA and reducing SB among children and adolescents may enhance their overall health status. Future research is needed to expand prospective cohort and intervention studies to address directionality and causality in the relationships between PA, SB and SRH among children and youth.

**Trial registration:**

PROSPERO - CRD42019142244. Registered on October 18, 2019.

## Background

Self-rated health (SRH) or self-perceived health captures a person’s perception of their overall health status, physical health and mental health and has been used as an indicator of health-related quality of life (HRQOL) [1, 2]. It is commonly measured by a single-item question with a 5- or 4-point Likert response scale indicating a graded level of health status (e.g., from “poor” to “excellent” health). SRH covers multiple aspects of health status, including general physical functioning, psychological health and health behaviours. Previous studies have shown that SRH is associated with a wide range of physical and mental health concerns and is an independent predictor of morbidity and mortality [3–7]. Prior research has also documented that SRH is a stable health outcome measure from adolescence to early adulthood [2, 7]. The use of self-perceived health as a global health indicator can provide insights into the effect of behavioural risk factors on overall health among children and youth. Assessment of child and youth’s self-perceived health is important to identify children and adolescents with poor health and to guide population health intervention programmes targeted improving children and youth’s health.

The health benefits of physical activity (PA) have been well established among adult populations [8, 9]. Among children and adolescents, it has been documented that PA is associated with both physical and mental health and HRQOL [10–14]. Promoting PA among children and adolescents is beneficial for childhood and adolescent obesity prevention as well as some chronic disease conditions (e.g., cardiovascular disease, depression) [10, 12, 15]. Sedentary behaviour (SB) is defined as “any waking behaviour characterized by an energy expenditure≤1.5 metabolic equivalents (METs), while in a sitting or reclining posture” [16]. SB is associated with an adverse health status in children and adolescents. The impaired health consequences from SB include but are not limited to physical disabilities, poor psychological health and mental health disorders (e.g., depression, anxiety) among children and adolescents [10, 17–20]. Over the last two decades, the rapid development of science and technology has led to the popular use of electronic media devices among adults, youth and children. Children and adolescents increasingly engage in watching television (TV), excessive use of smartphones, playing video games or computer games, resulting in excessive sedentary time and decreased time in physical and sports activities [17]. Therefore, it is important to study the impact of PA and SB on health among children and adolescents.

The relationship between PA, SB and self-rated health has been mostly investigated among adult and elderly populations, and the relationship of PA, SB with SRH is shown in a dose-response pattern [21–24]. In children and adolescents, the association between PA, SB and multidimensional HRQOL has been investigated [13]. Our previous systematic review study showed that an inactive lifestyle and higher sedentary time correlated with lower HRQOL, including overall HRQOL and the physical and mental components of HRQOL among children and adolescents [13]. Studies on the associations among PA, SB and SRH in children and adolescents have emerged during the last decade [1, 25–28]. However, to the best of our knowledge, no systematic review has comprehensively investigated the relationships between PA, SB and SRH among children and youth. Specifically, there is a lack of evidence concerning the nature of the relationship of PA, SB with SRH in terms of strength, dose-response and linearity. A systematic review in this field will help to provide a better understanding of the associations of SRH with health-related behaviours and is important to provide evidence-based recommendations for guiding population health programmes aimed at promoting active living and healthy lifestyles among children and adolescents.

The purpose of this systematic review was to synthesize associations between PA, SB and SRH in the general population of children and adolescents and to investigate the dose-response relationship between PA, SB and SRH.

## Methods

### Protocol and registration

This systematic review was registered with the International Prospective Register of Systematic Reviews (PROSPERO; Registration number: CRD42019142244), available from https://www.crd.york.ac.uk/prospero/display_record.php?RecordID=142244. We reported this review following the Preferred Reporting Items for Systematic reviews and Meta-Analyses (PRISMA) guidelines [29].

### Literature search

We conducted searches in the MEDLINE, PSYCINFO and EMBASE electronic databases for English literature published from 1946 to December 30, 2019. The database searches were initiated in October and November 2018, and the update search was conducted in February 2020. The medical subject headings and keywords used in the electronic database search included ‘physical activity’, ‘exercise’, ‘accelerometer’, ‘sedentary behaviour’, ‘screen time’, ‘television, or TV, or television viewing’, ‘computers’, ‘video games’, ‘lifestyle’, ‘quality of life’, ‘health status’, ‘self-rated health’, ‘self-perceived health’, ‘self-report health’, ‘children’, ‘adolescents’, ‘childhood’, ‘adolescence’, and ‘youth’. The detailed literature search strategy for the electronic databases and the number of retrieved records are provided in the Additional files (see Additional file 1). We searched PubMed and manually checked the references in the identified included studies and the relevant reviews and meta-analyses for additional eligible studies. We also executed Google Scholar searches to identify additional published articles and unpublished studies. The database searches were conducted by one author.

### Inclusion and exclusion criteria

The following inclusion criteria were adopted for selection of the eligible studies: (1) Studies used a single-item question representing self-rated or self-perceived health as the primary outcome. The response options were in the form of a Likert scale (from “poor” to “excellent” health). Self-rated health was defined as health reported by children or adolescents themselves or by parents for their children’s health when the children were less than 8 years old. (2) Study participants were drawn from schools or communities representing the general population of children and adolescents aged primarily between 3 years and 19 years. For longitudinal studies with a follow-up age greater than 19 years, the age range was applied to the baseline time point when the exposure measure was collected. (3) The study design included cross-sectional, cohort and case-control studies that examined the association between PA and/or SB and SRH. (4) Measures of the exposure included physical activity and sedentary behaviour. Both subjective and objective measures were included.

The exclusion criteria included (1) studies that examined associations between PA, SB and SRH among children and adolescents with specific chronic disease conditions (e.g., obesity or diabetes) or among adults; (2) publications that were reviews, meta-analyses, study protocols, conference abstracts and proceedings, non-peer-reviewed journal articles, comments, letters, case reports and guidelines.

The relevant reviews and meta-analyses were not included in the synthesis, but their reference lists were examined for identification of other eligible studies that were not found in the database search. As the literature search for unpublished studies identified no other unpublished studies except one thesis that met the inclusion criteria [30], we excluded the thesis in the review.

### Study selection

The retrieved citations from the database search were independently screened by two authors, XYW and TZH for selection of the studies. The authors screened the titles and abstracts of the references in accordance with the predefined inclusion and exclusion criteria. For potential qualified studies, the full-text articles were retrieved and then separately reviewed by the two authors to determine the eligibility for inclusion. The full-text articles of relevant reviews and meta-analyses were also retrieved for examination of the references. Disagreements regarding the eligibility of the studies for inclusion were resolved by discussion among all the authors.

### Data extraction

We used a standardized extraction form to collect data from individual studies for the synthesis. The extracted information included characteristics of the study (e.g., first author, country, publication year, study design, sample size, participants’ age and gender), assessments of the exposure and outcomes, statistical methods, main findings and risk of bias assessment for each study. The data for meta-analysis were extracted using a Microsoft Excel spreadsheet.

### Data synthesis

The extracted data from individual studies were narratively synthesized in summary tables, including the characteristics and key findings of each study. The statistics for the associations among PA, SB and SRH within a study included an odds ratio (OR) and the 95% confidence interval (CI) in a logistic regression or a regression coefficient and the 95% CI in a linear regression.

For those studies with quantitative data suitable for meta-analysis, we performed a meta-analysis to synthesize the overall associations between the exposure of interest and SRH. As most included studies utilised a logistic regression using a binary categorical SRH outcome (e.g., “poor” versus “good” health), we estimated the difference in the odds of poor SRH between a lower level and a higher level of PA. For the effect of SB, we estimated the difference in the odds of poor SRH between a higher level of SB (e.g., ≥2 h/day) and a lower level of SB (e.g., < 2 h/day). Subgroup meta-analyses were conducted by gender of the participants, PA dose level and type of SBs (TV viewing, use of computers and total screen time). To account for potential heterogeneity across studies, we used a random effects model in the meta-analysis. The Cochran Q and I^2^ statistics were used to test the degree of heterogeneity. A *p*-value less than 0.1 in the Q test and an I^2^ value greater than 50% indicated statistically significant and substantial heterogeneity, respectively [31]. To test the statistical significance of the dose-response relationship between PA and health status, we used a meta-regression, where the effects of both within- and between-study variances were accounted for. Publication bias was detected using a funnel plot and Egger’s test [32, 33]. The funnel plot asymmetry was tested by Egger’s test, in which the standardized effect (e.g., log odds) was regressed against its standard error (precision), with a *p*-value< 0.1 for the intercept (α) indicating a statistically significant asymmetry or a presence of publication bias [33]. The meta-analysis was conducted using Stata/SE 15.0 (Stators LLC, College Station, Texas, USA).

### Assessment of risk of bias

We used the *Quality Assessment Tool for Observational Cohort and Cross-Sectional Studies* (QATOCCS) provided by the US National Heart, Lung, and Blood Institute [34] to evaluate the risk of bias. The QATOCCS included 14 questions covering the following aspects: research question and study objective, population specification, participation rate, recruitment of participants, sample size justification, time of the exposure collection, time of study, exposure levels, validation of exposure assessment, outcome measures and blinding, loss to follow-up, and adjustment of potential confounding variables in regression analyses. Each question was assigned a score of one if a confirmative answer ‘yes’ was appropriate for the study. The total score was obtained by sum of the score for each question, ranging between zero and 14, with a higher total score for a study indicating low risk of bias. In reference to the previous research for categorization of the study quality level [17], a study was classified as high quality or low risk of bias (score 11–12), medium quality or moderate risk of bias (score 9–10), and low quality or potential high risk of bias (score 7–8).

## Results

### Characteristics of the included studies

**Fig. 1:**
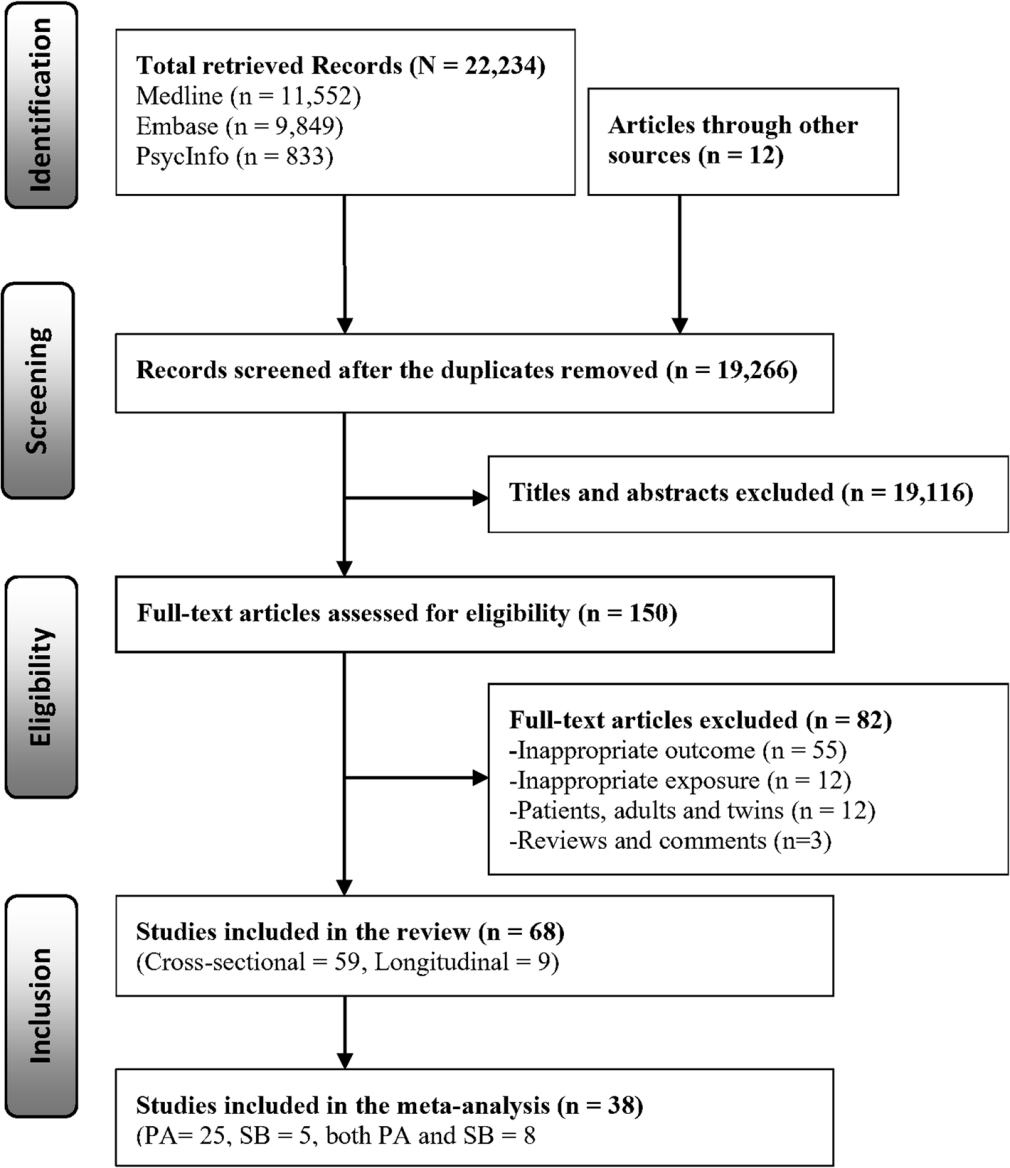
PRISMA flow diagram for selection of the included studies

We identified 22,234 citations through the electronic database search in MEDLINE (*n* = 11,552), EMBASE (*n* = 9849) and PSYCINFO (*n* = 833). An additional 12 articles were obtained through the reference list, PubMed and Google Scholar searches for related articles. After deleting duplicate records identified in the different databases (*n* = 2980), we screened 19,266 published records for eligibility through title and abstract review. Of these, 150 studies were retained for full-text evaluation, and 82 of them were then excluded due to ineligibility. Finally, 68 studies met the inclusion criteria and were included in the synthesis (Table 1). The study selection is presented in the PRISMA flow diagram (Fig. 1). The excluded references in the full text evaluation are presented in the Additional files (see Additional file 2).

**Table 1:** Sample characteristics and the key finding for the association between PA, SB and SRH of the included studies (*N* = 68)

First author, publication year and country	Sample ***n*** (% of girl)	Mean age (years) or age range	Associations		Risk of bias score
			PA and SRH	SB and SRH	
**Cross-sectional study**					
Marques, 2019 Portugal [35]	5024 (52.8)	13.9	P+	NS	9
Silva, 2019 Brazil [36]	6259 (59.7)	16.6	P+	–	9
Jodkowska, 2019 Poland [37]	1173 (Girls only)	15	P+	N-	8
Werneck, 2018 Brazil [38]	984 (58.8)	10–17	–	N-	9
Li, 2018 Japan [39]	4966 (50.7)	15.8	P+	N- for girls	9
Granger, 2017 European countries [40]	13,783	15	P+	NS	10
Lachytova, 2017 Slovak Republic [4]	1111	14–16	P+	N-	10
Matin, 2017 Iran [41]	13,486 (49.2)	12.47	P+	N-	10
Novak, 2017 Croatia, Lithuania and Serbia [27]	6501(52)	14–19	P+	–	10
Sharma, 2017 Peru [26]	1234 (61.4)	11–19	P+	–	10
Husu, 2016 Finland [25]	851	7–14	P+	N-	12
Koelmeyer, 2016 Australia [42]	2717 (Males only)	10–19	P+	–	7
Sharma, 2016 Peru [43]	970 (53.8)	14.5	P+	N-	10
Ustinavičienė, 2016 Lithuania [44]	1730 (49.8)	15.86 (boys), 15.81(girls)	–	N- for boys	8
Badura, 2015 Czech Republic [45]	10,503 (50.8)	11,13,15	P+	–	9
Herman, 2015 Canada [1]	7725 (49)	12–17	P+	N-	11
Kantomaa, 2015 Finland [46]	7063	16	P+	–	10
Martínez-López, 2015 Spain [47]	2293 (50.2)	14.2	P+	N-	11
Meireles, 2015 Brazil [48]	1042 (47.2)	11–17	P+	NS	9
Padilla-Moledo, 2015 Spain [49]	680 (46.0)	6–17.9	–	N-	9
Novak, 2015 Croatia [50]	3427 (50.7)	17–18	P+	–	9
Smith, 2015 UK [51]	3105	11–12	NS	NS	9
Chun, 2014 South Korea [52]	3676	16–18	P+	–	9
Craike, 2014 Australia [53]	732 (Girls only)	7–11	NS	–	8
Dyremyhr, 2014 Norway [54]	2510	15–20	P+	–	9
Herman, 2014 Canada [3]	527 (46.3)	9.64 (boys), 9.59 (girls)	P+ for boys	N- for girls (PC/video)	11
Kovacs, 2014 Hungary [55]	881 (44.6)	16.6	P+	–	8
Moor, 2014 28 European and North American countries [56]	117,460 (53.3)	11–15	P+	N-	10
Brooks, 2014 UK [57]	4404 (51.6)	11, 13, 15	P+	–	8
Afridi, 2013 Pakistan [58]	414 (46.1)	14.36	NS	–	9
Do, 2013 South Korea [59]	136,589 (47.7)	13–18	–	N-	11
Galán, 2013 Spain [60]	21,188	11–18	P+	–	11
Spein, 2013 Greenland and Norway [61]	728 (56.5)	15–16	P+	–	8
Richter, 2012 Germany [62]	6997 (49.9)	11–15	P+	–	10
Tabak, 2012 Poland [63]	600 (50.8)	13.2–13.7	P+	NS	7
Veloso, 2012 Portugal [64]	3069 (54.1)	14.8	P+	N-	9
Zullig, 2011 US [65]	245 (54.7)	11–15	P+	NS	7
Foti, 2010 US [66]	12,193	grade 9–12 high school	P+	N-	10
Iannotti, 2009 North America and Europe [67]	49,124	11,13, 15	P+	N-	11
Kahlin, 2009 Sweden [68]	1090 (57.1)	18.1	P+	–	10
Mathers, 2009 Australia [69]	925 (49.6)	16.1	–	N- for video games	9
Page, 2009a Thailand [70]	2492 (66.7)	16.2	P+	–	7
Page, 2009b Central and Eastern European [71]	3123	16.6	P+	–	9
Richter, 2009 European and North American countries [72]	97,721 (52.0)	13, 15	P+	N-	10
Breidablik, 2008 Norway [73]	2741	18.3	P+	–	11
Söderqvist, 2008 Sweden [74]	1269 (52.2)	15–19	–	N-	11
Kelleher, 2007 Ireland, Europe and North America [75]	123,653 (51.1)	9–18	P+	N-	8
Piko, 2007a Hungary [76]	1114 (60.1)	16.5	P+	–	10
Piko, 2007b Hungary [77]	548 (45.3)	12.2	P+	–	9
Alricsson, 2006 Sweden [78]	993 (51.0)	18.0	P+	–	7
Piko, 2006 Hungary [79]	1109	14–21	P+	–	7
Watanabe, 2006 Japan [80]	804 (48.9)	3–5	P+	–	9
Brodersen, 2005 UK [81]	4320	11.8	P+	NS	9
Honkinen, 2005 Finland [82]	994	12	P+	–	10
Erginoz, 2004 Turkey [83]	4153 (47.0)	16.4	P+	–	10
Pastor, 2003 Spain [84]	1038 (50.9)	16.31	P+	–	10
Tremblay, 2003 Canada [85]	12,715	12–17	P+	–	10
Vingilis, 2002 Canada [86]	1493	12–19	P+	–	10
Thorlindsson, 1990 Iceland [87]	1131 (49.0)	15–16	P+	–	9
**Longitudinal study**					
Burdette, 2017 US [28]	7827 (54.0), 14-year FU	15.76 (baseline)	P+	–	11
Liu, 2015 Japan [88]	5238 (51.8), 6-year FU	6 (baseline)	P+	–	11
Nigg, 2015 US [89]	334 (55.1) at FU, 5-year FU	14.76 (FU)	NS	NS	8
Spengler, 2014 Germany [90]	953 (54.5), 6-year FU	11–17 (baseline)	P+	NS	9
Bauldry, 2012 US [91]	10,375 (53.0), 12-year FU	15.47 (baseline)	P+	–	11
Elinder, 2011 Sweden [92]	2489 (51.8), 3-year FU	15.6 (baseline)	P+ for boys	–	11
JerdÊn, 2011 Sweden [93]	1046 (50.3), 2-year FU	12–14 (baseline)	P+	–	10
Breidablik, 2009 Norway [2]	2399, 4-year FU	13–19 (baseline)	P+	–	12
Sacker, 2006 UK [94]	29,470 (49.1), 15 to 17-year FU	16 (baseline)	P+	–	11

*PA* physical activity, *SB* sedentary behaviour, *SRH* self-rated health, *P+* positive association, *N-* negative association, *NS* not statistically significant association, − not applicable, *FU* follow up, *UK* United Kingdom, *US* United States

Table 1 presents the major characteristics of the included studies, the findings of the associations and the risk of bias assessment. The detailed results are presented in the Additional files, including the assessments of PA, SB and SRH, statistical methods and confounders adjusted for in the regression, and the key results (see Additional file 3). The review included 59 cross-sectional studies and nine longitudinal studies [1–4, 25–28, 35–94]. Most of the studies (*n* = 43) were conducted in European countries (e.g., UK, Spain, Finland, Sweden, Norway, Portugal, Hungary) or used data from multiple countries. The remaining 25 studies came from other countries, including the United States (*n* = 5), Canada (*n* = 4), Australia (*n* = 3), Japan (*n* = 3), Brazil (*n* = 3), Peru (*n* = 2), South Korea (*n* = 2), Iran (*n* = 1), Pakistan (*n* = 1) and Thailand (*n* = 1). The sample size of the studies varied between 245 (the smallest sample study) [65] and 136,589 (the largest sample study) [59]. Of the included studies, 24 studies examined both PA and SB for health status, 38 studies assessed the association between PA and SRH, and six studies analysed the effect of SB on SRH.

Most of the studies used self-report questionnaires to evaluate PA and SB. PA was measured by asking children or adolescents about their physical activities (type and intensity, duration and frequency) within the past 7 days or one month. SB was usually measured by time (hours or minutes) spent on sedentary behaviours (e.g., watching TV, playing video games, using computers, etc.) in a week or a day. Two studies used a device-measure of PA and SB (e.g., accelerometer) [3, 25]. Self-rated health was mostly categorized into two groups (“poor” versus “good” health) and analysed with logistic regression. Six studies used ordinal logistic or multinomial logistic regression models [2, 28, 73, 74, 86, 93]. Six studies used a linear regression treating SRH as a continuous variable [53, 63, 67, 81, 89, 91].

### Risk of bias assessment

Fifteen studies were rated as high quality or low risk of bias (score range 11–12), 39 studies were rated as medium quality or moderate risk of bias (score range 9–10), and 14 studies were classified as low quality or high risk of bias (score 7–8) (Table 1). The reasons for categorizing studies with a high risk of bias included a small sample or convenience sample, inadequate statistical analysis (e.g., not adjusting for confounding effects in regression analysis), or use of limited exposure levels of PA.

### Associations between physical activity and self-rated health

#### Findings of the included studies in the systematic review

Of the 62 studies that examined the association between PA and SRH, most studies (*n* = 58) showed statistically a significant positive association between PA and SRH (see Table 1). Only four studies did not observe a significant relationship between PA and SRH [51, 53, 58, 89]; three used a relatively small sample [53, 58, 89], and one investigated girls only [53].

A number of studies observed a dose-response relationship between PA and SRH, where an increasing level or amount of PA was related to a higher odds of “good or excellent” SRH (see Additional file 3) [1, 4, 27, 41, 42, 46, 48, 54, 60, 68, 70, 86]. For example, the study by Lachytova et al. (2017) found in a sample of adolescents aged 14–16 years old that relative to students who exercised less than once a week, students who exercised every day were 8.04 times more likely to report “good and excellent” health; students who exercised 4–6 times a week and 2–3 times a week were 3.67 times and 1.35 times, respectively, more likely than those exercised less than once a week to have “good and excellent” health after adjusting for gender, BMI, mental health and sedentary behaviour in the logistic regression [4]. Herman et al. (2015) reported that Canadian boys who were moderately active or inactive were 1.59 and 2.09 times, respectively, more likely to report lower health than peers who were physically active [1]. The regression adjusted for confounding effects of age, ethnicity, highest household education, smoking status, BMI and screen time. A similar result was observed for girls (adjusted OR = 1.31, 95% CI: 1.09–1.59 for moderately active versus active; adjusted OR = 1.99, 95% CI: 1.67–2.36 for inactive versus active).

Eight out of the nine longitudinal studies observed a significant positive association between higher PA and better SRH. For example, Breidablik et al. (2009) found in a 4-year follow-up prospective study in Norway that adolescents who insufficiently engaged in sports and exercise at baseline were more likely to have “poor” health at follow-up (adjusted OR = 1.64, 95% CI: 1.45–1.86) [2]. Liu et al. (2015) observed that children aged 6 years who maintained regular physical activity in outdoor PA during the 6-year follow-up had higher perceived health at follow-up than their peers who were physically inactive [OR (95% CI): 1.37 (1.17–1.60) for total sample; 1.45 (1.14–1.85) for boys; 1.23 (1.00–1.51) for girls] [88]. Sacker et al. (2006) reported in a large British cohort study (*n* = 15, 452) that a higher frequency of PA during adolescence predicted better SRH in their adulthood [94].

#### Meta-analysis results for PA

**Fig. 2:**
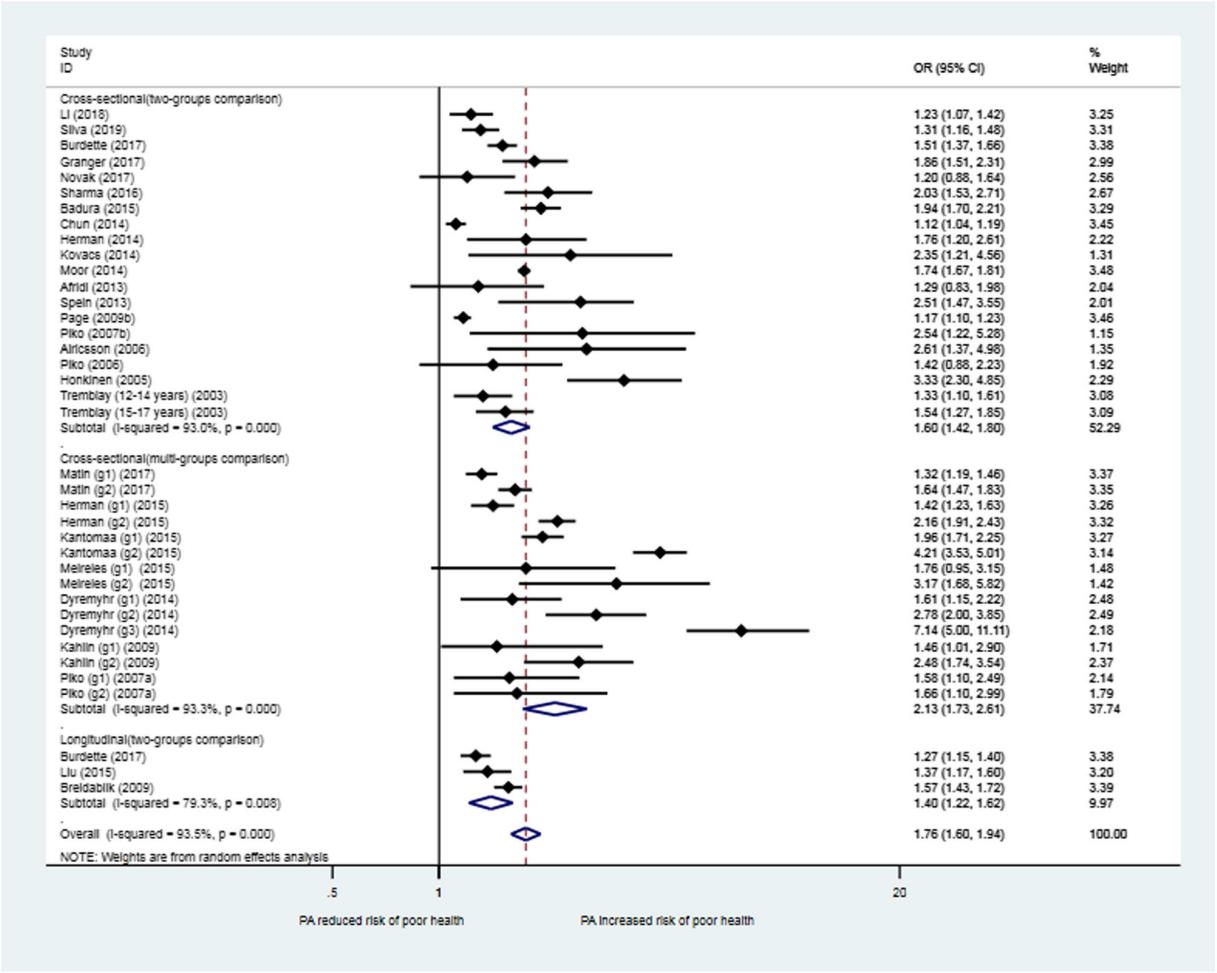
Forest plot for the association between physical activity and self-rated health in children and adolescents (total sample): OR (95% CI) for poor health comparing a lower level with a higher level of physical activity. Legend: Matin (g1-g2): g1-Low PA vs. Moderate PA, g2-Low PA vs. High PA; Herman (g1-g2): g1-Inactive vs. Moderate active, g2-Inactive vs. High active; Meireles (g1-g2): g1-Insufficiently active vs. Active, g2-Inactive vs. Active; Dyremyhr (g1-g3): g1-No PA vs. Small PA, g2-No PA vs. Moderate PA, g3- No PA vs. High PA; Kahlin (g1-g2): g1-Low PA vs. Moderate PA, g2-Low PA vs. High PA; Piko (g1-g2) (2007a): g1-PA Sometimes vs. Regularly, g2-No or occasionally PA vs. Regularly. Kantomaa (g1-g2): g1-Poor/Moderate health vs. Good health, g2-Poor/Moderate health vs. Very good health

Figure 2 shows the meta-analysis results for 28 studies that investigated the relationship of SRH with PA stratified by PA level. The unadjusted OR in the included studies was used in the meta-analysis. Together, 34 out of the 38 between-group comparisons among studies in the model showed a significant difference in SRH in favour of the higher PA groups. The estimated overall OR (for “poor” health) among all the included studies was 1.76 (95% CI: 1.60, 1.94) when comparing low PA to moderate or high PA. For those studies that examined more than two levels of PA (low/no PA versus moderate PA, low/no PA versus high PA), the combined OR was 2.13 (95% CI: 1.73, 2.61), indicating a dose-response effect of PA on health (e.g., higher PA was associated with better health). The studies in the meta-analysis showed high heterogeneity (I^2^ = 93.5%, *p* < 0.01), which may be explained by the differences in the measurement and categorization of PA across studies.

Meta-regression for seven studies with three exposure levels of PA in each study showed that the comparison between low and high PA groups was 77% more likely to report “poor” SRH (OR = 1.77, 95% CI: 1.16, 2.69) than the comparison between low and moderate PA groups, indicating a strong dose-response association (M 1, Table 2).

**Table 2:** Meta-regression analysis results for the effect of PA, SB on SRH: odds ratio (OR) and 95% confidence interval (CI) for poor SRH

Model	Comparison groups	OR	95% CI	***P*** value
M 1	**PA and SRH** (total sample, *n* = 15) (reference: Moderate PA)			
	Low vs. High PA	1.77	1.16, 2.69	**0.012**
	Low vs. Moderate PA	1.57	1.15, 2.13	**0.008**
M 2	**PA and SRH** (by gender, *n* = 32)			
	Girls vs. boys	0.88	0.66, 1.19	0.407
	Boys (reference)	1.84	1.49, 2.67	**< 0.001**
M 3	**SB and SRH** (total sample, *n* = 25) (reference: Total screen time)			
	Computers/video games	1.05	0.85, 1.30	0.630
	TV viewing	1.04	0.86, 1.27	0.676
	Total screen time	1.25	1.06, 1.47	**0.010**
M 4	**SB and SRH** (by gender, *n* = 18)			
	Girls vs. boys	1.07	0.92, 1.25	0.353
	Boys (reference)	1.15	1.04, 1.29	**0.013**

Bold values for *p* value in the table indicate statistical significance (*p* < 0.05)

**Fig. 3:**
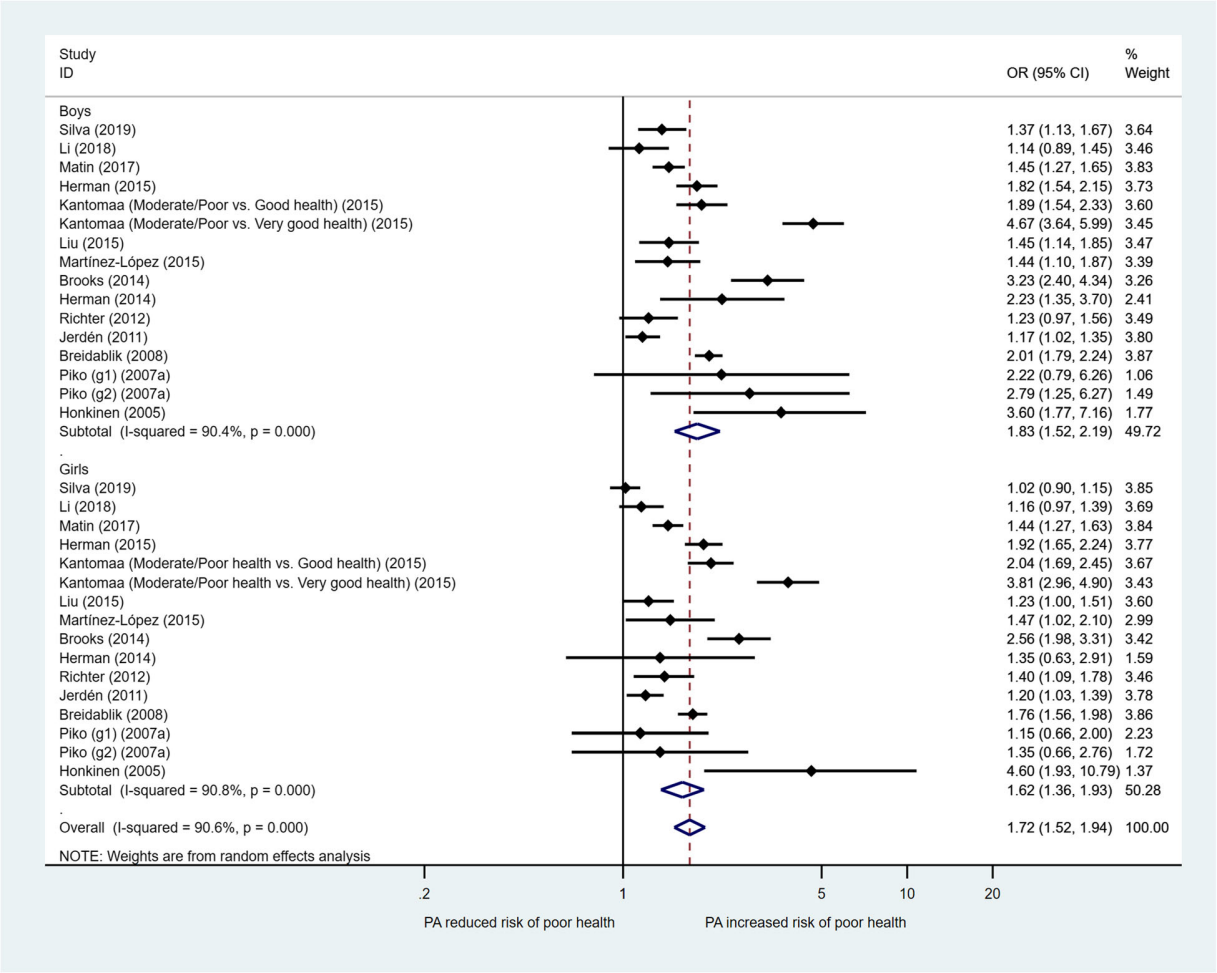
Forest plot for the association between physical activity and self-rated health in children and adolescents (by gender): OR (95% CI) for poor health comparing low level with higher level of physical activity. Piko (g1-g2) (2007a): g1-PA Sometimes vs. Regularly, g2-No or occasionally PA vs. Regularly. Other study groups compared PA Low level vs. Moderate or High level

Figure 3 presents the meta-analysis of the relationship of SRH with PA by gender. The pooled OR (95% CI) (for “poor” health) between low PA and high/moderate PA was 1.62 (1.36, 1.93) among girls and 1.83 (1.52, 2.19) among boys. Meta-regression showed no significant gender difference in the odds of “poor” SRH across PA levels (*p* = 0.407) (M 2, Table 2).

Egger’s test showed no significant risk of publication bias for the studies in the meta-analysis for the total sample (α = 1.53, t = 1.66, *p* = 0.11) or for the studies by gender (α = 1.71, t = 0.97, *p* = 0.35 for boys; α = 1.69, t = 0.95, *p* = 0.36 for girls). The funnel plots for PA and SRH are presented in Fig. A and Fig. B in the Additional files (see Additional file 4).

### Associations between sedentary behaviour and self-rated health

#### Findings of the included studies in the systematic review

Of the 30 studies that examined SB and SRH, 21 studies showed a statistically significant negative relationship between SB and SRH. Nine studies did not find a significant association [35, 40, 48, 51, 63, 65, 81, 89, 90]. Two studies observed a significant relationship between SB and SRH for girls only [3, 39], and one study found a significant relationship for boys only [44] (see Table 1).

The relationship between SB and SRH was observed for different types of sedentary behaviours, including watching TV, using computers or playing video games and total screen time. Lachytova et al. (2017) showed that adolescents who watched TV less than two hours a day were more likely to report “good and excellent” health (OR = 2.36, 95% CI: 1.35, 4.10) than their peers who watched TV two or more hours a day (see Additional file 3) [4]. Husu et al. (2016) used a device-measure of SB (accelerometer) and found that a one-hour increase in sedentary time a day was related to 29% lower likelihood of reporting “excellent” health relative to “good/fair/poor” health (OR = 0.71, 95% CI: 0.62, 0.82) among children after controlling for the effects of gender and school grade [25]. Herman et al. (2015) observed that adolescent with daily screen time greater than two hours had a higher odds of experiencing “poor” health in comparison with adolescent with daily screen time shorter than two hours (OR = 1.40, 95% CI: 1.19, 1.66 for boys; OR = 1.50, 95% CI: 1.30, 1.74 for girls) [1].

#### Meta-analysis results for SB

**Fig. 4:**
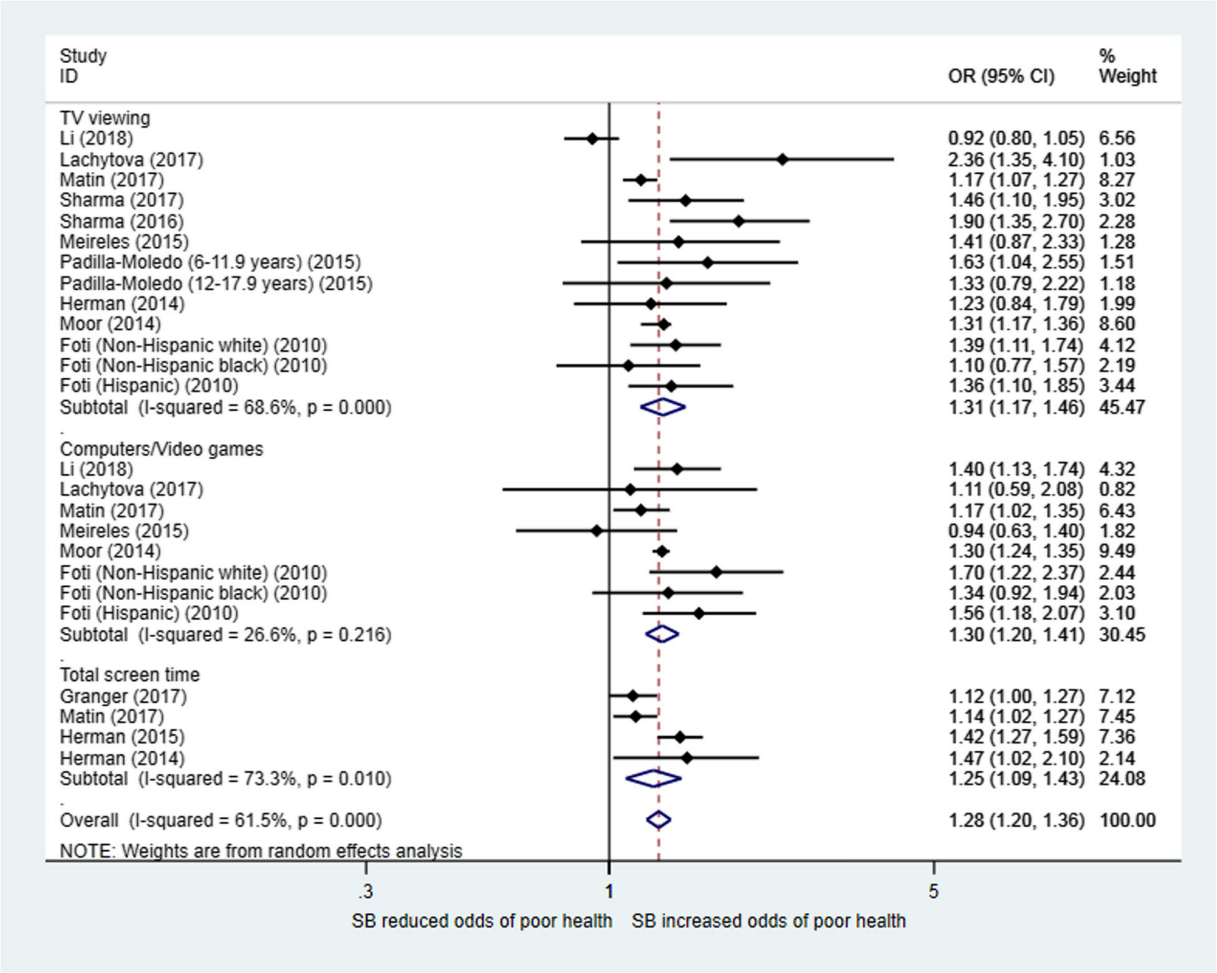
Forest plot for the association between sedentary behaviour and self-rated health in children and adolescents (total sample): OR (95% CI) for poor health comparing higher sedentary time with lower sedentary time. Sedentary time levels: Granger (2017): ≥4 h/day vs. < 4 h/day; Foti (2010): ≥3 h/day vs. < 3 h/day; All other studies’ comparisons: ≥2 h/day vs. < 2 h/day

Figure 4 shows the meta-analysis results for 11 studies that examined the relationship between SB and SRH. The estimated overall ORs (95% CIs) for “poor” SRH when comparing higher with lower SB were 1.31 (1.17, 1.46), 1.30 (1.20, 1.41) and 1.25 (1.09, 1.43) for TV viewing, playing computers or video games and total screen time, respectively. There was moderate heterogeneity across all studies in the meta-analysis (overall I^2^ = 61.5%, *p* < 0.01), and there was no significant heterogeneity between studies for playing computers and video games (I^2^ = 26.6%, *p* = 0.216). The meta-regression analysis did not show a significant difference in the odds of “poor” SRH among the three different types of SBs (Table 2).

**Fig. 5:**
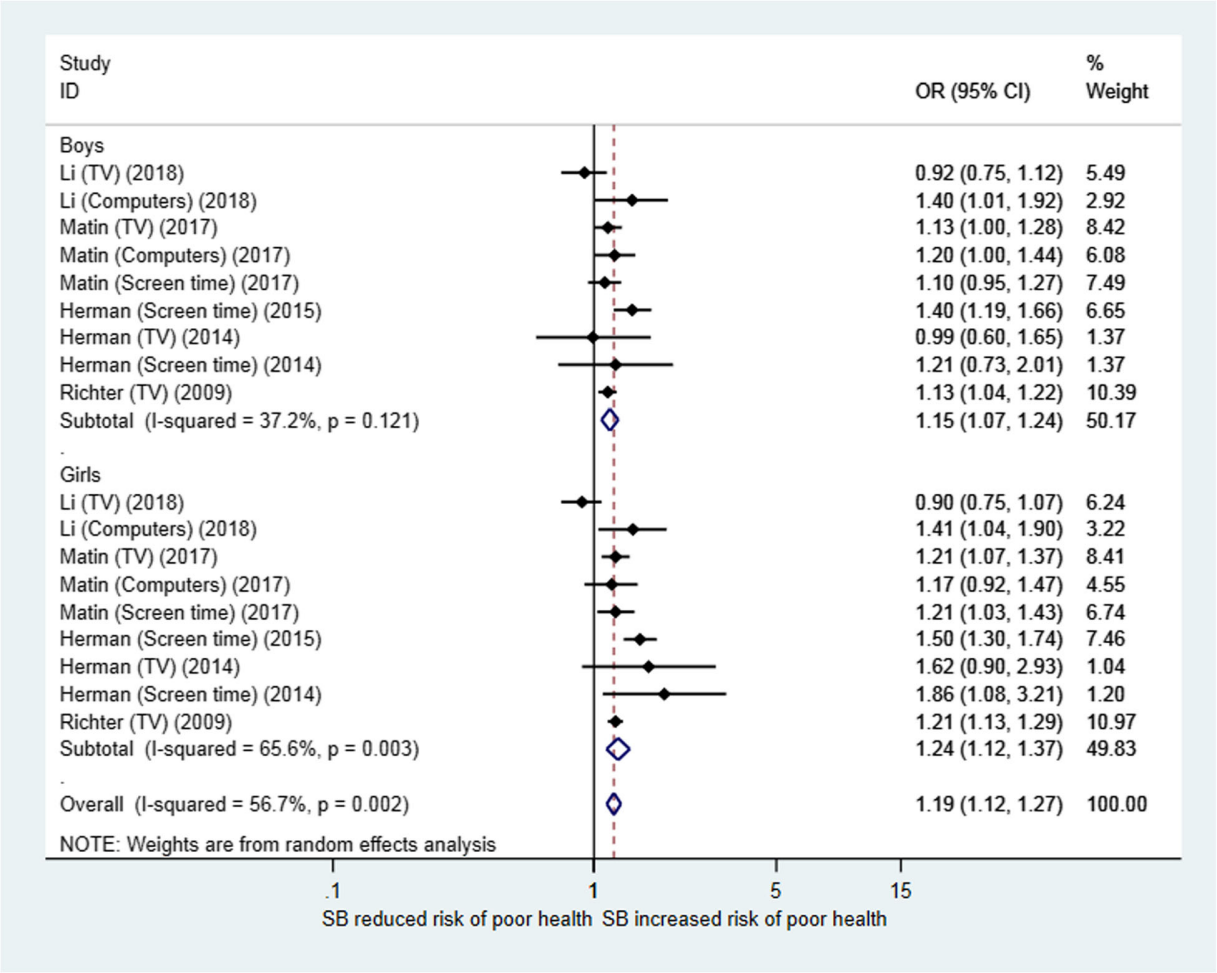
Forest plot for the association between sedentary behaviour and self-rated health in children and adolescents (by gender): OR (95% CI) for poor health comparing higher sedentary time with lower sedentary time. Sedentary time levels: Richter (2009): ≥4 h/day vs. < 4 h/day; All other study comparisons: ≥2 h/day vs. < 2 h/day

Figure 5 presents the meta-analysis results for the relationship between SB and SRH by gender. The combined OR (95% CI) (for “poor” health) between high and low SB was 1.15 (1.07, 1.24) for boys and 1.24 (1.12, 1.37) for girls. Meta-regression did not show a significant gender difference in the effect of SB on SRH (*p* = 0.353) (Table 2).

Egger’s test showed no significant risk of publication bias for the studies that investigated the effect of SB (*α* = 0.38, t = 0.77, *p* = 0.45 for the total sample; *α* = 0.26, t = 0.28, *p* = 0.79 for boys; *α* = 0.70, t = 0.65, *p* = 0.54 for girls). The funnel plots for SB and SRH are shown in the Additional files (see Fig. C and Fig. D in Additional file 4).

## Discussion

This systematic review found strong evidence for a positive relationship between physical activity and SRH and a negative relationship between sedentary behaviour and SRH among children and adolescents. The associations were observed in both cross-sectional and longitudinal studies. We found evidence of a significant dose-response association between PA and self-rated health. The observed associations between PA, SB and SRH appear independent of gender, age, body weight status, mental health and household socio-economic status among children and adolescents.

To the best of our knowledge, this is the first study that systematically synthesized the associations of SRH with PA and SB among younger populations of children and adolescents. Positive associations between PA and SRH and inverse associations between SB and SRH have been demonstrated among adults and older people [22, 24, 95, 96]. The finding in the present study is consistent with previous studies demonstrating positive relationships between PA and SRH among adults [22, 24, 95, 96]. Eight out of the nine included longitudinal studies found a positive association between PA and SRH, suggesting that PA among children and adolescents may predict future health status in adolescence and adulthood. The findings from this study add to the literature for detecting a dose-response association between increasing levels of PA and elevated perceived health among children and youth. The association appears to be in a log-linear increasing trend between PA levels and SRH, and this dose-response pattern was found for both boys and girls.

The present study found a consistent association between different forms of SBs (TV viewing, playing computers or video games and total screen time) and lower SRH among children and adolescents. Children and adolescents who spent more time on a SB (e.g., ≥2 h/day versus < 2 h/day) were more likely to experience poor SRH. There was no significant difference in the association by gender (Fig. 5) or by SB type. As most of the studies categorized SB in two levels (≥2 h/day versus < 2 h/day) in the regression analysis, we were not able to examine the dose-response effect by comparing more than two levels for SB using meta-analysis due to the sparse availability of data.

One of the innovative aspects of the present review is that we analysed both PA and SB exposures and included population-based studies with large samples. This enabled us to examine whether the effects of PA and SB on health outcomes were independent of each other and of other confounding variables. Since multivariable regression analyses adjusted for a number of confounders, such as demographics (e.g., gender, age), household economic factors, BMI, mental health problems and parental factors (e.g., education, smoking, health), the observed correlations among PA, SB and SRH can be considered robust regardless of gender, age, socio-economic factors, body weight status and mental health among children and youth. Some studies simultaneously adjusted PA and SB variables in the regression analysis [1–4, 26, 67, 72], hence, the effect PA and SB for SRH may be regarded as mutually independent.

Additionally, we detected the magnitude of differences in self-rated health between PA or SB comparison groups and found that the difference in some studies exceeded a minimally important difference (MID) value [97], defined as a clinically meaningful difference in health status that may signify a practical importance for modifying public health interventions among children and adolescents. The MID criterion varies across health status measures and the type of statistics for the association of interest. In terms of the effect size value using an odds ratio for a binary categorical outcome, prior research suggests that a log odds greater than 2.0 or less than 0.5 is considered a large effect size [98]. A number of studies reported an odds ratio above 2.0 or below 0.5 [1, 3, 4, 39, 43, 46, 54, 60, 68, 79, 82], suggesting a large effect size.

The findings in this study further reinforce the existing evidence by showing that a single-item question of self-rated health is a sensitive and valid indicator for general health in school-aged children and adolescents. Previous studies have demonstrated that poor SRH in adolescence is related to prescribed medication in adulthood [99] and is a risk factor for elevated morbidity and mortality [91, 100]. The observation in the present review strengthens the discriminative validity of the self-rated health reported by children and youth. Subject or patient self-reported outcome measures have been increasingly used to evaluate health status among general populations as well as patients with various diseases [9, 13]. The single item of SRH has been frequently used together with multi-component quality of life measures (e.g., the SF-36) to assess overall health or its relationship with physical and psychological health among adults [101] and is a useful tool in large population surveys to monitor the health of populations and study the effects of various socio-economic risk factors and health-related behaviours [95, 96]. This study highlights the utility of the single-item SRH among children and adolescents. Future research is encouraged to expand investigations on the role of SRH in predicting physical and mental morbidities among children and adolescents, as previously demonstrated among adults [5, 6].

Due to the heterogeneity in the measurement of PA and SB and in the statistical methods (e.g., the regression method), we performed meta-analyses for the studies with comparable data that used logistic regressions to quantify the associations between the exposures and health outcomes. We included a relatively large number of studies in the meta-analysis. For the studies reporting frequencies of SRH for different categories of PA and SB, we calculated the odds ratios and the confidence intervals for the synthesis in meta-analyses. A few studies used other statistical methods, such as linear regression, t-test or ANOVA and were not included in the meta-analysis due to the heterogeneity in statistics and small number of studies. Regarding the effect of PA, several studies used lower SRH as a reference group (e.g., coded as “good/excellent” versus “poor/fair” health) with more than two levels of PA in the logistic regression and were not included in the meta-analysis due to the difference in the grouping of PA (e.g., PA as clusters or days/week) [4, 27, 42, 45, 60].

The strengths of this review include a comprehensive literature search in both the published and grey literatures and stringent methodology adhering to the PRISMA statement, the inclusion of both PA and SB, the use of meta-regression analysis, and the inclusion of large sample studies with diversified socio-economic/socio-demographic backgrounds of children and adolescents. Population-based studies with large samples allowed to perform a multivariable regression analysis, enabling robust parameter estimates for inference to target populations. Large-sample studies yield narrower confidence intervals for the estimated parameters, thus providing more precise results than small-sample studies. Meta-regression analysis allowed us to test subgroup differences in health outcomes by gender, the PA dose level and the type of SBs among the participants. Additionally, the studies included in the present review were conducted in a wide range of countries and regions; hence, the findings in this review may be generalizable to broad regions in the world. While we made efforts to search and include grey literature, we did not include unpublished literature because only one thesis was identified as eligible. We do not expect that the grey literature would change the results, as previous studies have shown that the exclusion of unpublished studies and dissertations/theses had little influence on the estimates of health outcomes in a systematic review [102]. It is recommended that the inclusion of grey literature in a systematic review should be considered in those areas where there are very few published studies [102, 103].

This review is limited by the small number of longitudinal studies included, affecting the inference about the direction of the association between PA, SB and SRH. More longitudinal and prospective studies and intervention are needed to study the characteristics of this relationship. In addition, the assessment of PA and SB in the included studies was largely based on self-report, which is prone to be affected by measurement errors. Studies using device measures of PA and SB are required to assess more accurately the relationship between PA, SB and SRH among children and youth.

## Conclusions

This study found that higher PA is associated with better SRH and excessive SB is related to poor SRH in adolescents and children. The study reveals that there is a positive and dose-response association between PA and SRH in children and adolescents. These findings suggest that school-based programmes promoting active lifestyles and reducing SB may enhance the health status of children and adolescents. Public health policy and practice should prioritize interventions for both PA and SB tailored to children and adolescents with unhealthy behaviours to increase and maximize their health benefits.

## Supplementary information

**Additional file 1.** Literature search strategy used in the databases of MEDLINE, EMBASE and PSYCINFO.

**Additional file 2.** References of the excluded studies from the full-text review.

**Additional file 3.** Study characteristics, assessments of PA, SB and SRH, and the main findings.

**Additional file 4.** Fig. A–D funnel plots: comparisons of the odds of “poor” SRH by PA and SB.

## Data Availability

All data are available in the manuscript and in the supporting information.

## Abbreviations

SRH: Self-rated health
PA: Physical activity
SB: Sedentary behaviour
PRISMA: Preferred Reporting Items for Systematic reviews and Meta-Analyses
HRQOL: Health-related quality of life
OR: Odds ratio
CI: Confidence interval
