# Supplementary material for: Associations between physical activity, sedentary behaviour and self-rated health among the general population of children and adolescents: a systematic review and meta-analysis
Source: BMC Public Health. 2020 Sep 3;20:1343. doi: 10.1186/s12889-020-09447-1 (PMC7650260; doi:10.1186/s12889-020-09447-1)
Supplement: Supplementary file 1 — Additional file 1. Literature search strategy used in the databases of MEDLINE, EMBASE and PSYCINFO. [file 12889_2020_9447_MOESM1_ESM.doc]

**Additional file 1**

**Table Literature search strategy in the database of MEDLINE, EMBASE and PSYCINFO**

| **Database** | **Search terms** |
| --- | --- |
| **MEDLINE** | (1946 to December 30, 2019) |
| 1 | Physical activity OR Exercise* |
| 2 | Life style* OR sedentary lifestyle* OR sedentary behavior OR Health Behavior* |
| 3 | Video Games OR Television* OR screen time OR television viewing OR sitting time |
| 4 | Computer games OR Computers* |
| 5 | Accelerometer OR Pedometer |
| **6** | **1 OR 2 OR 3 OR 4 OR 5** |
| 7 | Health status* |
| 8 | Self-rated health OR Quality of life* OR self-perceived health |
| **9** | **7 OR 8** |
| 10 | Child* OR childhood OR Adolescent* OR Adolescence OR Teenager OR Youth |
| **11** | **6 AND 9 AND 10** |
| 12 | limit 11 to (english language AND humans AND journal article AND yr="1946 -Current") |
| **Total records retrieved: 11,552** | |
|  |  |
| **EMBASE** | (1974 to December 30, 2019) |
| 1 | Physical activity* OR computer* OR using computer OR video game* |
| 2 | television viewing OR television* OR screen time |
| 3 | Accelerometer OR sedentary lifestyle* OR exercise OR sedentary behavior OR lifestyle* OR sitting |
| 4 | Health behavior* OR diet* OR vegetable* OR fruit* OR dietary behavior OR dietary intake* |
| **5** | **1 OR 2 OR 3 OR 4** |
| 6 | Health status* OR self-rated health OR self-reported health OR Quality of life* OR self-perceived health |
| 7 | Child* OR adolescent* OR adolescence OR childhood OR youth OR juvenile* |
| **8** | **5 AND 6 AND 7** |
| 9 | limit 8 to (human AND english language AND journal AND yr="1946 -Current") |
| **Total records retrieved: 9,849** | |
|  | |
| **PSYCINFO** (1946 to December 30, 2019) | |
| 1 | Health Behavior OR DIETS * OR Physical Activity OR Exercise* OR Lifestyle* OR Sedentary Behavior* OR Physical Fitness* OR Eating Behavior* |
| 2 | Television viewing* OR television OR COMPUTERS* OR computer |
| 3 | Video games OR Computer Games* OR screen time * |
| **4** | **1 OR 2 OR 3** |
| 5 | Quality of Life* OR health status |
| 6 | self-rated health OR self-perceived health OR self-reported health OR self-report health |
| **7** | **5 OR 6** |
| 8 | Children OR adolescent OR teenager OR youth OR childhood OR adolescence |
| **9** | **4 AND 7 AND 8** |
| 10 | limit 9 to (human AND all journals AND english language AND yr="1946 -Current") |
| **Total records retrieved (PsycInfo): 833** | |
| **Total records retrieved from the three databases: 22,234** | |

*: exploded
