# Supplementary material for: Associations between physical activity, sedentary behaviour and self-rated health among the general population of children and adolescents: a systematic review and meta-analysis
Source: BMC Public Health. 2020 Sep 3;20:1343. doi: 10.1186/s12889-020-09447-1 (PMC7650260; doi:10.1186/s12889-020-09447-1)
Supplement: Supplementary file 2 — Additional file 2. References of the excluded studies from the full-text review. [file 12889_2020_9447_MOESM2_ESM.doc]

**Excluded references through the full-text review**
