# Supplementary material for: Associations between physical activity, sedentary behaviour and self-rated health among the general population of children and adolescents: a systematic review and meta-analysis
Source: BMC Public Health. 2020 Sep 3;20:1343. doi: 10.1186/s12889-020-09447-1 (PMC7650260; doi:10.1186/s12889-020-09447-1)
Supplement: Supplementary file 3 — Additional file 3. Study characteristics, assessments of PA, SB and SRH, and the main findings. [file 12889_2020_9447_MOESM3_ESM.doc]

**Table Characteristics, assessments of physical activity, sedentary behavior and self-rated health among children and adolescents, covariates in regression analyses and the main findings of the included studies**

| **Author, Year, Country** | **Sample (age)** | **PA and/or SB assessments** | **SRH assessment** | **Main findings** | | **Covariates in regression analyses** |
| --- | --- | --- | --- | --- | --- | --- |
|  |  |  |  | **PA and SRH** | **SB and SRH** |  |
| **Cross-sectional study** | |  |  |  |  |  |
| Marques et al., 2019 Portugal | Age range: 10–17, Mean age = 13.9 (CI:13.9-14.0) | PA was assessed as the number of days over the past week that adolescents were physically active for at least 60 minutes (min) per day. The response options were from 0 (none) to 7 (daily). Responses were dichotomized into≤6 times per week and daily. SB included watching TV, playing video games and using computers. Total screen-based time was calculated by the sum of the three SBs, and was dichotomised into ≥2 h and <2 h daily. | Self-perception of health question: “You would say your health is…?”, 4-point scale of responses: poor, fair, good and excellent. | P+  Mean score (95% CI) of SRH by PA: PA≤6 times/wk: 3.2 (3.2, 3.2); Daily PA: 3.5 (3.5, 3.6), p<0.001. | NS  Mean score (CI) of SRH by screen-based time:  ≥2 h/day: 3.2 (3.2, 3.3); <2 h/day: 3.2 (3.2, 3.3), p = 0.426. | t-test |
| Silva et al., 2019 Brazil | Age range: 14–19, Mean age = 16.6 (SD1.3) | PA questions: “how many days the adolescents were physically active for a total of at least 60 minutes a day, and over the last 7 days or during a typical or normal week”. PA<5 days was considered insufficiently active. | Self-perceived health question with the response options: bad, regular, good and excellent. The variable was categorized as: Excellent/good health, and Regular/poor health. | P+  Insufficiently active (vs. active), unadjusted prevalence ratio (PR) (95% CI): 1.37 (1.13–1.67) in boys; 1.02 (0.90–1.15) in girls. Adjusted PR (CI): 1.27 (1.04–1.56) for boys; 0.99 (0.88–1.3) for girls. | --- | Age, maternal schooling, place of residence. Preferred leisure type, weight status. |
| Jodkowska et al., 2019 Poland | Age range: 15 | Moderate to vigorous PA (MVPA) was assessed by asking girls how many days they were physically active for a total of at least 60 min per day in the past 7 day. It was dichotomized as MVPA≥7days/week, and <7days/wk. Vigorous PA (VPA) question: “outside of school hours, how often do you usually exercise in your free time so much that you get out of breath or sweat?” The responses were dichotomized as VPA 2–3 times a week or more, and VPA below 2–3 times a week. SB was assessed by the number of hours a day that girls usually spent in free time on watching TV, using computers, videos, DVDs, and other entertainment on a screen. SB variable was categorised as ≥2 h/day, and <2 h/day. | The SRH question: “Would you say that your health is? With 4 options: excellent, good, fair and poor. The variable was grouped as high SRH (excellent/good) and low SRH (fair/poor). | P+  UOR(CI) for high SRH: MVPA (≥7days/wk vs. <7 days/wk): 1.66 (1.12–2.45); VPA (≥2–3 times/ wk vs. <2–3 times/wk): 1.50 (1.23–2.03); Participation of PE classes (Regular vs. Irregular): 1.82 (1.40–2.38).  AOR(CI): VPA: 1.39 (1.06–1.78); Participation of PE classes (Regular vs. Irregular):1.57 (1.18 –2.08). | N-  UOR(CI):  TV/DVD (≥2 h/day vs. <2 h /day): 1.38 (1.08–1.79);  Using social media (≥2 h/day vs. <2 h /day): 1.50 (1.17–1.93). | Chronic diseases, diet-related variables, amount of sleep on school days. |
| Werneck et al., 2018 Brazil | Age range: 10–17 | Adolescents screen time was assessed by asking about how many hours a day were spent watching TV, using computer and video games on weekdays and weekends. The mean hour of screen time was used for each SB. | SRH question: “In general, how do you consider your health?” Responses: 4-point Likert scale ranging from bad (= 1) to excellent (= 4) health. | --- | Coefficient (B):  For boys: -0.158,  p = 0.015; For girls: -0.188, p = 0.007. | Structural equation modeling (SEM).  Mediators: Distress, peer and family relationships. |
| Li et al., 2018 Japan | Age range: 15–16, Mean age = 15.8 (SD0.3) | PA measure included four response categories: doing PAs nearly every day, ≥3 times per week, 1–2 times per week, and <1 time per week. Sedentary lifestyles included time of video game playing (0, 0–1 h, 1–2 h, 2–3 h, >3 h for one day), telephone usage (0, 0–0.5 h, 0.5–1 h, >1 h for one day); TV viewing, use of mobile phone e-mail and personal computers (≤2 h, 2–3 h, 3–4 h, >4 h for one day). | The healthquestion: “In the past 4 weeks, how did you assess your health?”, with five response options: excellent, very good, good, fair, and poor. The outcome was classified as good health (excellent/very good/good) and poor health (fair and poor). | P+  PA≤2 times/wk vs. ≥3 times/wk), UOR (95% CI): Boys: 1.14 (0.89−1.45). Girls 1.16 (0.97–1.38). | NS for SBs among boys. N-for using PC among girls.  Girls: use of PC>4h/day (vs.≤2 h/day): UOR (CI): 2.83 (1.39–5.76); AOR (CI): 2.24 (1.06–4.72). | Breakfast eating, night time food intake, night time sleep duration. |
| Granger et al., 2017 European countries | Age: 15 (SD1.0) | PA was defined as number of days that participants were physically active for a minimum of 60 minutes per day over the last 7 days. Participants who achieved the required PA level on all 7 days were assigned to the ‘active group’. Participants who only achieved 60 min of PA on 6 days or fewer were classified as the ‘inactive group’. SB was assessed by hours a day watching TV or using a computer or a video game, and analyzed with two levels: ≥4 h/day, <4 h/day. | A 5-point Likert rscale for SRH: excellent, very good, good, fair or poor health. The responses were combined into two groups: good health (excellent/ very good/good) and poor health (fair or poor). | P+  Inactive group vs. active group, UOR (CI): 1.861 (1.513–2.29); AOR (CI): 1.607 (1.245–2.074). | NS for SB  SB <4 h/day vs. ≥4 h/day, UOR (CI): 1.124 (0.998–1.267); AOR (CI): 1.101 (0.952–1.274). | Gender, BMI, socioeconomic status and sedentary behaviour |
| Lachytova et al., 2017 Slovak Republic | Age range: 14–16 | PA question: “How often do you do sports (such that you breathe hard and sweat) in your free time?” Response options: every day, 4–6 times a week, 2–3 times a week, once a week, once a month, less than once a month and never. SB: Average time on watching TV or using a computer on a school day: less than 2 hours, and ≥2 hours. | SRH was from the Short Form Health Survey (SF-36), with 5 response options: excellent, very good, good, not bad and bad. The variable was dichotomized as good/excellent health (options 1, 2 and 3) and less than good health (not bad and bad). | P+  (Reference group: less than once a week), UOR (CI) for ‘good/excellent health’: PA every day: 11.53 (2.02–65.78), 4–6 times/wk: 4.23 (1.20–14.90), 2–3 times/wk: 2.42 (0.77–7.54), Once a week: 1.40 (0.43–4.58).  AOR (CI): PA every day: 8.04 (1.62–39.85), 4–6 times/wk: 3.67 (1.14–11.78), 2–3 times/wk: 1.35 (0.57–3.18), Once a week: 0.94 (0.35–2.55). | N- for TV; NS for using PC.  TV<2 (vs. ≥2 h TV): UOR (CI) for good/excellent health: 2.36 (1.35–4.10); AOR (CI): 2.56 (1.37–4.79). | Gender, BMI, mental health problems, PA, SB. |
| Matin et al., 2017 Iran | Age range: 6–18, Mean age = 12.47 (SD3.36) | PA was estimated by two questions: (1) Number of days of PA over 30 minutes a week (0–7days); (2) Time spent in physical education classes every week (0–3 h or more). PA less than 2 hours per week was considered as low, 2–4 hours a week as moderate and more than 4 hours a week as high PA. Screen time was assessed by time spent on watching TV and using computers. Prolonged screen time was defined as screen time for more than 2 hours a day. | Self-rated general health was categorized in two groups: poor and good. | P+  Reference group: Low PA:  UOR (CI): Moderate: 1.31 (1.18–1.46), High: 1.64 (1.45–1.85). AOR (CI): Moderate: 1.16 (1.03–1.31), High: 1.37 (1.20–1.57). | N-  For good (vs. poor) health, Screen time (reference: <2 h/day), UOR (CI): High: 0.87 (0.78–0.97). AOR (CI): 1.03 (0.91–1.16). | Age, sex, region, socioeconomic status, BMI, passive smoker, active smoker, anxiety, depression, body image, birth order. |
| Novak et al., 2017 European countries: Croatia, Lithuania and Serbia | Age range: 14–19 | Number of days of moderate-to-vigorous PA per week: (1) 0 days, (2) 1–2 days, (3) 3–4 days, (4) 5–6 days, (5) 7 days. Participants who had not participated in at least 300 min/week in the PA were categorized as insufficiently active, compared with those of participants with ≥300 min/week (sufficiently active). | A five-level Likert scale for health status: very poor, poor, fair, good and excellent. The answers were categorized as poor (very poor and poor) and good health (fair, good and excellent). | P+  (1) Total time of PA (Reference group: <300 min/wk): UOR (CI)): ≥300 min/wk: 1.20 (0.88–1.64). AOR (CI): 0.96 (0.69–1.34).  (2) Days of MVPA (reference group: 0 days): UOR (CI): 1–2 days: 2.13 (1.62–2.79), 3–4 days: 2.18 (1.62–2.93), 5–6 days: 2.19 (1.44–3.33), 7 days: 2.37 (1.30–4.35). AOR(CI):  1–2 days: 1.78 (1.34–2.38), 3–4 days: 1.79 (1.30–2.45), 5–6 days: 1.86 (1.12–2.75), 7 days: 2.04 (1.07–3.89). | --- | Age, gender, BMI, socioeconomic status, parent-child trust, neighbourhood trust, informal social control, teacher-student or student interpersonal trust, collaboration between students, psychological distress. |
| Sharma et al., 2017 Peru | Age range: 11–19 | PA was measured by the number of days of PA in the past week for no less than 60 min (0–7 days). SB was measured by time (hours) spent on watching TV with the options: <1 h,1–2 h, 3–4 h, 5–6 h, 7–8 h and >8 h a day. For logistic regression analysis, students who watched TV more than 2 hours a day was considered as high viewing. | Five-level Likert scale for health status: excellent, very good, good, fair and poor. Poor and fair options were coded as poor health and the remaining options as good health. | P+  (Reference group: PA≥5 days).  AOR (CI):PA<5 days: 1.49 (1.12–1.98). | N-  (Reference group: TV≤2 h/day), AOR (CI): TV>2 h/day: 1.38 (1.03–1.85). | Sex, age group, PA, TV viewing, school and grade. |
| Husu et al., 2016 Finland | Age range: 7–14 | Objective measure of PA. Participants wore an accelerometer to measure PA and SB continuously for 7 days. Participants must use accelerometers for at least four days, at least 10 hours a day. PA was categorized into three intensity categories based on metabolic equivalents (MET): light PA: 1.5−2.9 MET; moderate PA: 3.0–5.9 MET and vigorous PA: 6 MET and over. | Self-perceived health with the options: excellent, good, fair and poor. | P+ for Excellent (vs. good/fair/poor) health.  (1) AOR (CI) adjusted for gender and school grade: Light PA (1 h increase/day): 1.88 (1.27–2.77), MVPA (min per day/10): 1.08 (1.03–1.13).  (2) Adjusted for all: Light PA (h/day): 1.69 (1.08–2.65), MVPA (min per day/10): 1.05 (1.00–1.11). | N-  AOR (CI), SB increase (h/day): (1) Adjusted for gender and school grade: 0.71 (0.62–0.82). (2) Adjusted for all: 0.77 (0.66–0.89). | Gender, school grade, BMI and health-related symptoms. |
| Koelmeyer et al., 2016 Australia | Age range: 10–19 | PA level in the past week: sedentary: 0 hours; insufficiently active: less than 60 min per day for 10–17 years old; sufficiently active: at least 60 min per day for 10–17 years old. | The question: “In the past four weeks, in general, how was your health?” Response options: bad, fair, good, very good and excellent. A dichotomous variable for ‘good to excellent’ health vs. ‘bad and fair’ health was used. | P+ for Excellent/good health, Sedentary as reference group.  UOR (CI): Insufficiently active: 1.05 (0.67–1.65), Sufficiently active: 1.98 (1.25–3.13). AOR (CI): Insufficiently active: 0.88 (0.53–1.47), Sufficiently active: 1.42 (0.84–2.40). | --- | Socioeconomic characteristics, fruit consumption, BMI, current smoker, excessive alcohol consumption, depression. |
| Sharma et al., 2016 Peru | Age range: 11–19, Mean age = 14.5 (SD1.6) | PA: number of days of PAs at least one hour per day in the past week. Responses of ‘less than 3 days’ were categorized into inactive and ‘3 or more days’ into active. Leisure time use had three groups: Internet, TV, Outdoor activity. | A five-point Likert scale for SRH, being dichotomous: ‘good’ (excellent, very good or good health) and ‘poor/fair’ (poor or fair health). | P+  Inactive vs. active, AOR (CI): 1.49 (1.03–2.15). | N-  TV watching during leisure time (vs. outdoor activity), AOR (CI): 1.70 (1.09–2.67). | Sex, age, family factors (meals skipping, fruit consumption, family support), lifestyles (smoking, alcohol, school absenteeism), life satisfaction, body weight. |
| Ustinavičienė et al., 2016 Lithuania | Age range: 13–18, Mean age (boys) = 15.86 (SE0.94), (girls) = 15.81 (SE0.93) | SB measured as hours of playing computer games every day in last month, with the options of <5 h and ≥5 h. Internet addiction measured as yes or no. | The responses for SRH were categorized into two groups: (a) excellent, very good and good; (b) poor and very poor. | --- | N- for SB for boys only  AOR (CI): Computer games (≥5 h vs. <5 h): 1.51 (0.94–2.43) for girls; 0.67 (0.41–1.12) for boys. Internet addiction (Yes vs. No): 2.48 (1.33–4.62) for boys; 1.53 (0.82–2.88) for girls. | Age, computer game type, time spent gaming per day, internet addiction. |
| Badura et al., 2015 Czech Republic | Age range: 11,13,15 | PA was measured by 6 items for organized leisure-time activities, including team sports, individual sports, art school, youth organizations, recreation/leisure centres, church meeting). The question asked “In your free time, do you do any of these organized activities?” with response categories yes or no. Clusters of these PAs were derived using cluster analysis, with six clusters: Active, All-rounders, Artists, Individual sports, Team sports and Inactive. | The question for SRH: “Would you say your health is …?”, with 4 response levels: excellent, good, fair and poor. A dichotomous variable was used (excellent health vs. good/fair/poor health). | P+  (1) ≥1 Activity vs. Inactive for Excellent health, UOR (CI): 1.94 (1.70–2.21); AOR (CI): 1.93 (1.69–2.20).  (2) PA clusters (reference: inactive), UOR (CI): All-rounders: 1.70 (1.47–1.96), Artists: 1.74 (1.49–2.04), Individual sports: 2.50 (2.11–2.95), Team sports: 2.21 (1.89–2.58). | --- | Age and gender |
| Herman et al., 2015 Canada | Age range: 12–17 | Respondents were asked about their participation in 21 specified activities and 3 additional volunteered activities (as frequency in the past 3 months and average session duration). Average daily energy during leisure time PA was calculated, weighting activities by their MET (metabolic equivalent of task) values. A PA index was categorized as active (≥3.0 KKD, kilocalories per kilogram per day), moderately active (1.5–2.9 KKD) or inactive (<1.5 KKD). Screen time was assessed by the time (hours) spent (in a typical week in the past 3 months) on playing computer games and using the internet (excluded time spent at work or at school), playing video games and watching TV or videos. Total screen time was categorized to 2 groups using the cut-point of 2 h/day. | SRH with 5 options (excellent, very good, good, fair or poor) was divided into two categories: (1) good, fair or poor, and (2) excellent or very good. | P+  UOR (CI), Active PA as the reference: Boys: Moderately active: 1.69 (1.42–2.00), Inactive: 2.14 (1.80–2.54); Girls: Moderately active: 1.36 (1.13–1.63), Inactive: 2.14 (1.81–2.52).  AOR (CI): Boys: Moderately active: 1.59 (1.33–1.90), Inactive: 2.09 (1.75–2.50); Girls: Moderately active: 1.31 (1.09–1.59), Inactive: 1.99 (1.67–2.36). | N-  Screen time >2 h/day vs. ≤2 h/day, UOR (CI): Boys: 1.40 (1.19–1.66); Girls: 1.50 (1.30–1.74). AOR (CI): Boys: 1.28 (1.08, 1.52); Girls; 1.32 (1.13–1.54). | Age, race/ethnicity, highest household education, smoking status, BMI, PA and screen time. |
| Kantomaa et al., 2015 Finland | Age range: 16 | PA question “How many hours a week all together do you participate in (a) brisk, and (b) light physical activity outside school hours?” The brisk PA reflects moderate-to vigorous PA. The adolescents were also asked about their daily time spent in physically active commutes to and from school. The PA level was converted into metabolic equivalent of task (MET) hours a week based on the intensity and the amount of these activities. MET hours were categorized into tertiles: (1) high (highest tertile), (2) average (middle tertile), and (3) low (lowest tertile). | SRH question: “How would you describe your health at the moment?”, with 5 responses: very poor, poor, fair, good, and very good. Three groups were given based on the responses: very good, good, and moderate/poor (fair, poor and very poor). | P+  UOR (CI)s were showed here.  (1) Good vs. Moderate/poor health: Boys: Average (middle tertile) vs. low PA: 1.60 (1.27–2.02); High (the highest tertile) vs. low PA: 2.43 (1.87–3.18). Girls: Average vs. low PA: 1.73 (1.40–2.12; High vs. low PA: 2.50 (1.99–3.15).  (2) Very good vs. Moderate/poor health: Boys: Average vs. low PA: 2.94 (2.24–3.88); High vs. low PA: 7.72 (5.74–10.38). Girls: Average vs. low PA: 2.70 (2.05–3.55); 5.48 (4.11–7.30). | --- | Obesity, parental socioeconomic position, smoking, emotional/behavioral problems, long-term illness, handicap, disability. |
| Martínez-López et al., 2015 Spain | Age range: 12–16, Mean age = 14.2 (SD1.3) | Two items asking PA (moderate-to-vigorous) at least one hour a day in the previous week and a typical week. The response options were “no, one day, two days, three days, four days, five days, six days, and seven days. A mean of the summed responses to both items was used and dichotomized as inactive or low PA level (1–5) and active or high PA level (6–8). SB: six items indicating the number of hours a day of watching TV, using PC , doing homework on weekdays and on weekends. The response included 9 options from 0 hour, half an hour, one hour, two hours…… to seven hours. The responses were classified as <2 h/day, 2–3 h/day and ≥4 h/day. | Responses included poor, reasonable, good, and excellent health; and were dichotomized as low-to-average (poor, reasonable and good) and excellent health. | P+  AOR (CI): Low (≤4 days/wk) vs. High (>4 days/wk) PA level: Boys: 1.436 (1.102–1.871). Girls: 1.467 (1.023–2.103). | N- for PC among boys, NS for TV.  SB≥4 h/day vs. <4 h/day, AOR (CI): PC on weekdays: Boys 1.217 (1.022–1.45), Girls 1.014 (0.825–1.248); PC on weekends: Boys 0.932 (0.796–1.092), Girls 0.941 (0.777–1.139). | Age and BMI. |
| Meireles et al., 2015 Brazil | Age range: 11–17 | PA over the last seven days (Active: 300 min or more and Inactive/Insufficiently active: up to 299 min).  SB: time spent per day watching TV, playing video games or computers (less than 1 hour, 2 hours; or 3 hours or more). | SRH with five options: very good, good, reasonable, poor or very poor. The responses were dichotomized into Poor (very poor, poor and reasonable) and Good (very good and good). | P+  AOR (CI): Inactive vs. Active; 1.43 (0.89–2.30); Insufficiently active vs. Active; 2.31 (1.15–4.69). | NS for watch TV and play video games or use computer. | Age, socioeconomic status, social support, fruits consumption, life satisfaction, BMI. |
| Padilla-Moledo et al., 2015 Spain | Age range: 6–17.9 | SB: TV viewing recorded as hours per day (6-point scale: none, <0.5 h, 0.5–2 h, 2–3 h, 4 h, >4 h). Participants were grouped into two levels: 0–2 h as low level, and >2 h as high level of SB. | Perceived health status was assessed by a 3-point scale: excellent, good and fair. | --- | N-  AOR (CI) for excellent health, TV >2 h vs. ≤2 h: Children (6–11.9 years): 0.614 (0.392–0.960); Adolescents (12–17.9 years): 0.753 (0.450–1.260). | Sex |
| Novak et al., 2015 Croatia | Age range: 17–18 | Total PA in the past 7 days was measured using the short version of the International Physical Activity Questionnaire (IPAQ). PA was expressed as metabolic equivalent-hours per week. | SRH with five options: very poor, poor, fair, good and excellent. The outcome was dichotomized as Good (excellent, good and fair) and Poor health. | P+ for Good SRH  AOR (CI), Low PA vs. High PA: 0.66 (0.42–1.04). | --- | Gender, BMI, socioeconomics, psychological distress, school and social capital variables. |
| Smith et al., 2015 UK | Age range: 11–12 | PA and SB were measured by the self-reported Youth Physical Activity Questionnaire (Y-PAQ). The questionnaire assesses the accumulated time spent physically active or sedentary respectively over the previous 7 days outside of school. The total time (hours per week) spent physically active in recreational games and sports, and the total time in sedentary activities (including screen time) outside of school was used. | The SRH variable was dichotomized to ‘fair/poor/very poor’ and ‘good/very good’. | NS  Mean score difference in mean hours (CI) of PA between fair/poor and good/very good health: 0.25 (-1.09, 1.58). | NS  Mean difference in hours (CI) of sedentary activities between fair/poor and good/very good health: 1.85 (-0.18, 3.88). | Gender, ethnicity, birth country, borough, parental employment, family affluence, neighbourhood amenities, aesthetics, walkability and safety. |
| Chun et al., 2014 South Korea | Age range: 16–18 | PA was measured by the question "How often did you exercise for more than 30 minutes during the past week?" with the responses: Never, Once per week, Twice per week, Three times per week, Four times per week, Five times and over. | Self-perceived health with four options: very poor, poor, good, very good. A dichotomous variable (very poor/poor and good/very good) was used. | P+  More exercise is related to higher odds of good and very good perceived health. AOR (CI): 1.115 (1.045–1.190). | --- | Age, gender, family economic, body weight, smoking, drinking, sleep, psychological factor, self-esteem, school, family, parents’ concern, community, school level factors. |
| Craike et al., 2014 Australia | Female children, Age range: 7–11 | PA was self-assessed using a single item indicating number of days (0–7) out of the past 7 days that adolescents engaged in PA for a total of one or more hours per day. | Responses on a 5-point scale ranging from excellent, very good, good, fair, to poor health. | NS  Linear regression for PA: B = 0.06 (CI: -0.007 to 0.065), p = 0.12. | --- | Year level, intrinsic/identified/introjected/external regulations, amotivation and competence. |
| Dyremyhr et al., 2014 Norway | Age range: 15–20 | Amount of PA during a week was classified as four levels: No PA, 0.5–1 hour (small amount), 2–4 hours (moderate amount), and 4 or more hours (great amount). | SRH question "How healthy do you think you are?" with the responses ‘very healthy, quite healthy and not very healthy’. A dichotomous variable was created (combined very healthy and quite healthy to one group). | P+  PA reference: no PA during a week  UOR (CI): Small amount: 0.62 (0.45–0.87); Moderate amount: 0.36 (0.26–0.50); Great amount: 0.14 (0.09–0.20). AOR (CI): Small amount: 0.44 (0.28–0.68); Moderate amount: 0.30 (0.19–0.47); Great amount: 0.12 (0.07–0.21). | --- | Gender, age, BMI, PA and type of sports. |
| Herman et al., 2014 Canada | Age range: 8–10, Mean age = 9.64 (SD0.97), 9.59 (SD1.03) for boys, girls respectively | PA and sedentary time were measured with the ActiGraph 7164 accelerometer. Children wore the accelerometer during all waking hours for 7 days. Light, moderate, and vigorous PA (LPA, MPA, VPA) were defined by the ActiGraph cut-offs (counts per minute, cpm): LPA: 100 cpm–2296 cpm, MPA: 2296 cpm–4012 cpm, VPA≥ 4012 cpm. Total PA (TPA) was the sum of LPA, MPA and VPA. Sedentary time was defined as time (in minutes) ≤100 cpm. SB: participants were asked about time (hours per day) spent in watching TV, playing computers/video games, doing homework, reading books. Daily hours of TV and computer/video games were summed to represent mean daily total screen time. All SBs were dichotomized: ≥2 h/day vs. <2 h/day. | SRH question: “In general, is your health excellent, mostly good, or not very good?” A dichotomous variable was used: excellent health vs. mostly good/not very good health. | P+ for boys  AOR (CI), MVPA <60 min/day vs. ≥60 min/day: Boys 2.23 (1.31–3.79), Girls 1.02 (0.47–2.23).  MVPA lowest tertile vs. High (upper 2 tertiles): Boys 6.10 (3.25–11.48), Girls 0.91 (0.50–1.65). | N- for girls for computer/video.  AOR (CI), Computer/video>2 h/day vs. ≤ 2 h/day: Boys 1.33 (0.78–2.24), Girls 2.24 (1.06–4.72). | Age, BMI, MVPA, SED. |
| Kovacs ea al., 2014 Hungary | Age range: 14–18, Mean age = 16.6 (SD1.3) | Participants were asked how many times they participated in at least 30 minutes of PA in the past 3 months. A new variable for PA was created and recoded as: no activity (never); irregular activity (a few times/2–3 times a month); and regular activity (1–2 times a week/3 or more times a week). | SRH was measured with a 4-point Likert scale: poor, fair, good, and excellent. The variable was recoded to two categories: poor/fair, good/excellent. | P+  OR (CI), Regular activity vs. Never: 2.35 (1.21–4.56). | --- | Not reported |
| Moor et al., 2014  28 European and North American countries | Age range: 11–15 | MVPA was measured, and coded as: 0 = At least 60 min on at least 5 days, 1 = Fewer days. SB included: watching TV, playing computer games, using PC (emails, homework, etc): <2 h and ≥2 h daily. | SRH with the response categories: excellent, good, fair, poor. The response options were dichotomized into excellent/good vs. fair/poor. | P+  AOR (CI), Fewer days activities vs. At least 60 min on at least 5 days: 1.74 (1.67–1.81). | N-  AOR (CI), TV≥2 h vs. <2 h: 1.31 (1.17–1.36). Computer games ≥2 h vs. <2 h: 1.30 (1.24–1.35). | Age and gender |
| Brooks et al., 2014 UK | 11, 13, 15 | The PA question: “Over the past 7 days, on how many days were you physically active for a total of at least 60 minutes per day?” Vigorous exercise during leisure activities outside of school (VEL) was measured using the HBSC question: “Outside school hours, how often do you normally exercise in your free time so much that you get out of breath or sweat?” Responses for PA were grouped into low (0–2 days), medium (3–6 days) and high (7 days). Responses for VEL were divided into low (once a week or less), medium (a few times per week) and high (every day). | SRH was divided into good/excellent versus other categories. | P+  Adolescents with medium or high PA were more likely to rate their health as good or excellent, with a difference between high and low PA of 22% for boys and16% for girls (Chi-square test, p<0.001). For VEL, the number of adolescents with good and excellent health was 20% (for boys) and 16% (for girls) higher in high VEL than low VEL group (p<0.001). | --- | Chi-square test |
| Afridi et al., 2013 Pakistan | Age range: 14–17, Mean age = 14.36 (SD1.08) | Physical inactivity was defined as moderate to intense activity<30 minutes for less than 4 days in past 7 days. In the analysis, PA was coded as two groups: 0–2 days/week, ≥3 days/week. | Students were asked if they were satisfied with their health with binary options of yes or no. | NS  UOR (CI), PA 0–2 days vs. ≥3 days: 1.29 (0.83–1.98). AOR (95% CI): 1.23 (0.78–1.95). | --- | Use of tobacco, diet, betel nuts, current and passive smoking. |
| Do et al., 2013  South Korea | Age range: 13–18 | SB was measured as the average daily use of internet (non-study purposes) over the past 30 days. Responses were grouped into weekdays and weekends. The sum of the time of weekdays and weekends was then divided by 7 to represent the average daily amount of time for internet use and was categorized into sextiles: 1st (0–17 min), 2nd (21–56 min), 3rd (60–86 min), 4th (90–124 min), 5th (129–180 min), 6th(184–630 min). | Question for SRH: “What do you think of your health status compared with your friends?” The options were: very unhealthy, unhealthy, neutral, healthy and very healthy. | --- | N- for Very healthy outcome  Average Internet use (sextile): coefficient (CI), 1st as reference): 2nd: -0.18 (0.24 to 0.12), 3rd: -0.18 (-0.24 to -0.13), 4^th^: -0.26 (-0.32 to -0.21), 5^th^: -0.32 (-0.38 to -0.27), 6^th^: -0.41(-0.47 to -0.34). | Gender, father’s and mother’s education, household economic status, type of residential area, school grade and survey year. |
| Galán et al., 2013 Spain | Age range: 11–18 | MVPA was assessed by the number of days of physical activity over 60 min in a week. The response categories were from 0 to 7 days. | SRH included 4 response levels: excellent, good, fair or bad. The health was categorized into optimal (excellent or good), and suboptimal health status (fair or bad). | P+  AOR (CI): Never PA (reference), for boys: 1–2 days: 2.37 (1.56 –3.58), 3–4 days: 2.34 (1.44–3.81), 5–6 days: 4.60 (2.60–8.13), 7 days: 4.05 (2.38–6.89). For girls: 1–2 days: 1.20 (0.85–1.69), 3–4 days: 1.51 (1.06–2.14), 5–6 days: 2.28 (1.47–3.52), 7 days: 2.14 (1.37–3.34). | --- | Sociodemographic and socioeconomic status (e.g., age, smoking, alcohol, BMI, fruit and vegetables), SB (e.g., watching TV/playing computers), academic achievement, household type and number of family members, etc. |
| Spein et al., 2013 Greenland and Norway | Age range: 15–16 | In the NAAHS (Norwegian Arctic Adolescent Health Study), PA was assessed by out of school activities: “how many times per week do you take part in sport/do physical exercise to the extent that you get out of breath or sweat?”, with the answer 0,1,2,3,4,5, and above. In the WBYG (Well-being among Youth in Greenland), participants were asked how often they exercised hard (running, football, etc.), with the responses: every day, at least once a week, less than weekly, never. A combined PA was categorized as two levels: (1) Frequent (one or more times a week or everyday), (2) Seldom (0 times a week, less than weekly or never). | SRH had 4 options: poor, not so good, good and very good in the NAAHS, and 5 responses: very poor, poor, fair, good and very good in the WBYG. A dichotomous outcome was used: Poor (good/not so good/poor) vs. Good in the NAAHS, and Poor (fair/poor) vs. Good in the WBYG. | P+  UOR (CI): For Sami: Frequent PA vs. Seldom: 0.17 (0.06–0.50); For Inuit: 0.41 (0.27–0.63). AOR (CI): For Sami: Frequent PA vs. Seldom: 0.21 (0.07–0.63); For Inuit: 0.50 (0.31–0.81). | --- | Adjusted for all other socio-demographics, risk- and protective correlates (e.g., suicidal behavior, alcohol use, smoking, well-being in school). |
| Richter et al., 2012 Germany | Age range: 11–15 | PA was assessed using a 60-min MVPA screening measure. PA (in past 7 days and a typical week) was classified into two groups: physically active for at least 60 min a day for at least 5 days or more versus fewer than 5 days. | The answer for SRH: Excellent, good, fair and poor. Responses were dichotomized as ‘Excellent/Good’ and ‘Fair/Poor’. | P+  AOR (CI), PA<6 days vs. 6–7 days: Boys 1.23 (0.97–1.56), Girls 1.40 (1.09–1.78). | --- | Age |
| Tabak et al., 2012 Poland | Age range: 13.2–13.7 | Adolescents were asked to report the number of days over the past week that they had been physically active for at least 60 minutes per day (MVPA). They reported how many hours per day spent on watching TV/videos/DVDs and using a computer (for playing games, emailing, chatting or surfing the internet) in their spare time on weekdays and weekends. Total screen time (hours/day) during the week was calculated. | The SRH question: “Would you say your health is...”, with response options of excellent, very good, good, fair, and poor. | P+  Rural: linear regression β = -0.14 (SE0.03), p<0.001; Urban: β = -0.06 (SE0.03), p<0.05. | NS  TV: β = 0 (SE0.01), p>0.05 for both rural and urban; PC: β = -0.01 (SE0.01) p>0.05 for both rural and urban. | Gender, PA, SB |
| Veloso et al., 2012 Portugal | Age range: 13–16.9, Mean age = 14.8 (SD1.1) | PA was measured by the number of days a week (1–7) doing 60 min of MVPA; intense exercise out of school (sport or leisure) was measured by frequency (never, <once a month, once a month, once a week, 2–3 times a week, 4–6 times a week, every day) and by hours a week (≥7 h, 4–6 h, 2–3 h, 1 h, 1/2 h, none). All SBs, including playing video games, computer and internet, TV, doing homework were measured by an average of hours/week. Three clusters were derived with the lifestyle variables of PA and exercise, screen time and eating behaviours (weekly consumption of fruits/vegetables, soft drinks, and sweets): *Active gamers (25%), Healthy group (41%) and Sedentary group (*34%). | The perceived health was measured by a 4-point scale: poor, fair, good, excellent. | P+  β = 0.15 (Active gamers vs. Healthy group); β = 0.11 (Healthy group vs. Sedentary group); β = -0.22 (Sedentary group vs. Active gamers). | N-  The sedentary group had poor health perception (β = -0.22) than healthy group. | NA (use of ANOVA) |
| Zullig et al., 2011 US | Age range: about 11–15 | PA was measured by the number of days (0, 1 or more) attending vigorous exercise, physical education classes in past days, a sports team (yes/no). PA was categorized into two groups: 0 days/week, ≥1days. SB was measured by watching TV in an average school day with two categories: less than 1h, 1 h or more. | Students described their health status with five responses: excellent, very good, good, fair and poor. SRH was dichotomized into ‘Fair/Poor health’, and ‘Excellent/Very Good/Good health’. | P+ for Sports team participation, NS for Vigorous PA.  Sports team participation vs. Not participation: OR (CI): Boys 5.40 (1.30–22.39), Girls 30.92 (3.74–255.43). Vigorous PA vs. no vigorous PA: OR (CI): Boys 5.91 (0.91–38.31). Girls 2.89 (0.53–15.61). | NS  TV watching ≥1 h vs. < 1 h/day: OR (CI): 2.42 (0.29–20.20) for boys, 0.51(0.14–1.85) for girls. | Not report |
| Foti et al., 2010 US | Adolescents (grade 9–12 high school students) | Types of PAs and the levels: (1) meeting PA recommendation levels during the seven days before the survey (yes/no); (2) playing on a sports team during the 12 months before the survey (yes/no); Attending physical education classes in school: attended daily, attended but not daily, did not attend PE classes. SB included: (1) watching TV: ≥3 h per day, <3 h per day; (2) Using computers: ≥3 h per day, <3 h per day. | SRH question had five response options: excellent, very good, good, fair and poor. The options were collapsed into three categories: excellent, very good/good, and fair/poor. | P+  AOR (CI): Not meet the recommended PA level vs. met the recommended level: Non-Hispanic white: 2.11 (1.76–2.52), Non-Hispanic black: 1.90 (1.40–2.58), Hispanic: 1.49 (1.08–2.05). Not play on a sports team vs. play on a sports team:  Non-Hispanic white: 2.59 (2.24–2.99), Non-Hispanic black: 1.69 (1.30–2.19), Hispanic: 1.78 (1.26–2.53). | N-  AOR (CI): TV≥3 h/day vs. <3 h/day: Non-Hispanic white: 1.39 (1.11–1.74), Non-Hispanic black: 1.10 (0.77–1.57), Hispanic: 1.36 (1.10–1.85). Computers≥3 h/day: Non-Hispanic white: 1.70 (1.22–2.37), Non-Hispanic black: 1.34 (0.92–1.94), Hispanic: 1.56 (1.18–2.07). | Sex and grade. |
| Iannotti et al., 2009 Ten countries of North America and Europe | Age range: 11,13, 15 | PA: students reported the number of days (1 to 7) they engaged in MVPA at least 60 mins over the past 7 days. Screen-based media use (SBM) was assessed by the number of hours (0h, 0.5 h, 1–7 h or more) per day (weekday and weekend) on using a computer during free time (excluding time spent doing homework) and watching TV (including videos). The mean hours per day of both screen-based activities were calculated and summed to create a SBM score. | Self-perceived health was rated with 4-point scale: fair, poor, good, and excellent. | P+  Linear regression coefficients (B): North America: 0.26; Western Europe: 0.11; Eastern Europe: 0.12; Northern Europe: 0.25; Southern Europe: 0.17 (p<0.0001 for all five regions). | N-  Screen-Based Media Use (B): North America: -0.07 (p<0.0001); Western Europe: -0.10 (p<0.0001); Eastern Europe: -0.02; Northern Europe: -0.09 (p<0.0001); Southern Europe: -0.02. | Age, gender, PA and SBM in the model. |
| Kahlin et al., 2009 Sweden | Age range: 16–26, Mean age = 18.1 | PA questions: “How often do you perform PA at a high level of effort (e.g. with increased pulse rate, breathlessness, sweating etc)? How often do you perform PA at a moderate level of effort? How often do you perform PA at a low level of effort?” The response options for the questions included: never, occasionally, once weekly, twice weekly, three times or more weekly. | SRH question: “How do you rate your general health?” with the options: poor, moderately poor, neither good nor poor, moderately good, very good. | P+  PA high level vs. low level: OR (CI): 2.481(1.739–3.539); PA moderate level vs. low level: OR (CI): 1.456 (1.013–2.901). | --- | Not report |
| Mathers et al., 2009 Australia | Age range: 13–19, Mean age = 16.1 (SD1.2) | Electronic media use of time data were collected by the Multimedia Activity Recall for Children and Adolescents (MARCA) that linked to energy expenditure. Minutes (per recall) spent on TV viewing, using a computer, playing video games and telephone use were calculated. | SRH with the responses: excellent; very good, good, fair, poor. The variable was dichotomized into ‘Excellent/Very Good’ vs. ‘Good/Fair/Poor’. | --- | N- for Video game use, NS for Total media use, TV, Telephone and computer use.  AOR (CI): video game use Low (0 to <45 min) (vs. No): 0.74 (0.52–1.05); High (vs. No): 1.61(1.13–2.29). | Sex, age, socioeconomic indexes for areas disadvantage index quartile, adolescent BMI z score. |
| Page et al., 2009a Thailand | Mean age = 16.2 (SD 1.33) | Four PA items were summed to a PA summary measure (Physical Activity Index). Types of PA reported in the table: vigorous PA, muscle strengthening activity, Number of sports teams played, activity index. | Subjects were grouped on the basis of their responses to SRH: very healthy, healthy, or not healthy. | P+  Vigorous PA, Mean (SD): Boys: Very healthy: 4.8 (1.9), Healthy: 3.6 (2.0), Not healthy: 2.5 (2.1) (p<0.0001). Girls: Very healthy: 2.9 (1.8), Healthy: 2.3 (1.7), Not healthy: 1.6 (1.5) (*P* <0.0001).  Activity index, Mean (SD):  Boys: Very healthy: 13.5 (5.0), Healthy: 10.4 (5.3), Not healthy: 7.2 (5.3) (p<0.0001). Girls: Very healthy: 8.5 (4.4), Healthy: 6.6 (4.2), Not healthy: 3.4 (3.5) (p<0.0001). | --- | NA (use of ANOVA) |
| Page et al., 2009b Central and Eastern European | Mean age = 16.6 | PA included days of participating in vigorous PA (making sweat and breathing hard for at least 20 min) during the past week, days of muscle-strengthening activity, number of sports teams playing on in past 12 months, amount of time involved in PA outside of school, and a summary measure of PA. | SRH was assessed by asking "How healthy would you say that you are?" with the response options: very healthy, healthy and not healthy. | P+  OR (CI): Vigorous PA: 1.17 (1.10–1.23), Muscle strengthening activity: 1.03 (0.98–1.08), Number of sports teams played on: 1.01 (0.98–1.06). | --- | Not report |
| Richter et al., 2009  33 European and North American countries | Age range: 13, 15 | PA was assessed with a 60 min MPVA screening measure, with two questions: “on how many days in the past week and in a typical week students were physically active for 60 min or more.” The scores of the two items were summed; a score of five or more days were classified as meeting the primary recommendation of more than one hour of moderate activity per day on most days. SB was assessed by asking “how many hours students usually watched TV (including videos) on weekdays and the weekend”. The responses were ‘none at all’, ‘about half an hour a day’, ‘about 1 h a day’, ‘about 2 h a day’ up to ‘about 7 or more hours a day’; both items were combined into one variable representing the average hours of TV watching per day. The responses were recoded into ‘4 h or more’ vs. ‘<4 h’. | SRH with the responses: excellent, good, fair, poor. Responses were dichotomized into ‘Excellent/Good’ vs. ‘Fair/Poor’. | P+  AOR (CI): Low PA (<5 days/wk) vs. high PA:  Boys: 1.87(1.70–2.05); Girls: 1.33 (1.21–1.46). | N-  AOR (CI): TV≥4h (vs. <4 h): Boys: 1.13 (1.04–1.22); Girls: 1.21 (1.13–1.29). | Age, family affluence, smoking, PA, TV use, breakfast, fruits and vegetables. |
| Breidablik et al., 2008 Norway | Age range:16.7–20.9, Mean age = 18.3 | PA included the following problems: Outside school, how many days a week do you play sport, or exercise to the point where you breathe heavily and/or sweat? Outside school, how many hours a week do you play sport, or exercise to the point where you breathe heavily and/or sweat? Are you actively involved in sports? | SRH question: “How is your overall health at the moment?”, with four options: very good, good, not very good and poor. ‘Poor’ and ‘Not very good’ were combined into one category. | P+ for Good/very good SRH, Ordinal logistic regression. Sports and exercise, UOR (CI): Boys: 2.01 (1.79–2.24); Girls: 1.76 (1.56–1.98). AOR (CI): 1.94 (1.71–2.21); Girls: 1.59 (1.38–1.84). | --- | Somatic health complaints, medical diagnosis, disability, family break-up, health services, mental health, self-esteem, general well-being, body concern, school relationship, health-compromising habits. |
| Söderqvist et al., 2008 Sweden | Age range: 15–19 | SB measures: TV viewing time (<30 min per day, 30–60 min per day, 60–180 min per day, >180 min per day); Time playing computer games (never, <30 min per day, 30–60 min per day, 60–180 min per day, >180 min per day); Use of wireless telephones such as mobile and cordless phones (DECT): Never, <5 min per day, 5–15 min per day, 15–30 min per day, >30 min per day; Use of mobile phone: <2 min per day, 2–5 min per day, 5–15 min per day, 15–30 min per day, 30–60 min per day, >60 min per day. | Respondents' perception of health during the last two months was collected with the options: very good, good, quite good, poor and very poor. | --- | N-  AOR (CI): Mobile phone use 2–15 min/day: 1.1 (0.8–1.4), 15–30 min/day: 1.4 (0.9–2.1), >30min/day: 1.5 (0.97–2.3). | Ordinal logistic regression.  Age and gender, insufficient sleep and tiredness. |
| Kelleher et al., 2007 Ireland, Europe and North America | Age range: 11, 13, 15 | PA was assessed by regular exercise (Infrequent vs. frequent exercise). SB measured by excess TV watching (more than 4 hours daily). | Respondents rated their health as very, quite or not very healthy, and this was dichotomized into Very vs. Quite or Not very healthy groups. | P+  Infrequent exercise, AOR:  Ireland male: 4.97, female: 2.86; International male: 2.34, female: 1.82. | N-  Excess TV viewing, Ireland sample: AOR=1.58 for boys and 2.10 for Girls. | Daily smokers, psychosomatic symptoms, bullied, relationship with friends, intoxicated, life satisfaction, exercise. |
| Piko et al., 2007a Hungary | Age range: 14–21, Mean age = 16.5 (SD1.3) | PA was measured by the exercise beyond school physical education for at least a half hour with the options from never to three or more times per week. The sports activity variable was classified into three categories: regularly on a weekly basis, sometimes/once or twice a month, no extra sport or just occasionally. | SRH was measured by asking respondents how they compared their health status to that of their peers, with four response options: poor, fair, good, excellent. The variable was dichotomized as ‘poor/fair’ vs. ‘good/excellent’. | P+  Reference group: sports activity regularly on a weekly basis. UOR (CI): Sport sometimes/once or twice a month: 1.58 (1.10–2.49) for total, 2.22 (0.79–6.26) for boys, 1.15 (0.66–2.00) for girls;  No extra sport or just occasionally: 1.66 (1.10–2.99) for total, 2.79 (1.25–6.27) for boys, 1.35 (0.66–2.76) for girls. | --- | Not report |
| Piko et al., 2007b Hungary | Age range: 10–15, Mean age = 12.2 (SD1.2) | Sports activity was measured by a question: ‘How many times in the last 3 months have you exercised (for at least half an hour)?’ Response categories included ‘not even in school, no extra sport just in school, occasionally, once or twice a month, once or twice a week, and three or more times a week. | Respondents were asked to rate their health status compared to their peers with four answer options from poor to excellent. A dichotomous variable was used: ‘poor/fair’ vs. ‘good/excellent’. | P+  Reference group: sports activity regularly on a weekly basis, UOR (CI): Sometimes/once or twice a month: 2.68 (0.57–12.73), No extra sport or just occasionally: 2.50 (1.10–5.80). | --- | Not report |
| Alricsson et al., 2006 Sweden | Age range: 16–19, Mean age = 18.0 (SD1.0) | Students reported how often they performed PA at a high, medium, or low level of effort. A high level was defined as increased pulse rate, breathlessness, and sweating. A medium level was defined as being able to talk with somebody during the physical performance, and a low level was defined as walking and/or cycling at a slow tempo. Total PA was categorized as two groups: Medium/high, Never/irregular/low. | SRH was based on the following three questions: (1) How do you judge your general health? (2) How do you rate your health? (3) How do you find your general health when compared with other individuals of the same age? | P+  Low level of PA is associated with poor SRH (p<0.001 in the Chi-square test); Correlation r = 0.219 (p<0.01). | --- | Chi-square test |
| Piko et al., 2006 Hungary | Age range: 14–21 | PA was measured by how many times the students exercised for at least half an hour in the past three months, with the response categories: never, once or twice, two or three times a month, once or twice a week, and three or more times per week. The variable was dichotomized into two categories: less active group (never or just occasionally) and regularly active group (minimum two or three times a month). | SRH was measured by asking respondents how they compared their health status to their peers. The responses included: poor, fair, good and excellent. | P+  Low PA is related to poor self-perceived health (p<0.01). | --- | Chi-square test |
| Watanabe et al., 2006 Japan | Age range: 3–5 | PA was measured by hours of outdoor play. | Child health conditions included two questions answered by their parents. The general health (GH) during the past 12 months was rated as “poor, fairly poor, fair and “good”. The question for sick days: “how often did your child have some symptoms of illness but did not visit a clinic? Responses were ‘never’, ‘one or two times’ and ‘frequently’. Child health conditions were classified into three categories in combination with the two questions: Good health (good GH with no sick days); Poor health (fair GH with frequent sick days and poor or fairly poor GH); Moderate health (one or two times of sick days). | P+  AOR (CI): Hours of outdoor play, Good health vs. Moderate health: 1.19 (1.03–1.37), Poor health vs. Moderate health: 1.05 (0.83–1.33)**.** | --- | Sex, age, mother's factor, father's education, family income, housing type, rented or not. |
| Brodersen et al., 2005 UK | Age range: 11–12, Mean age = 11.8 | PA was measured by the number of days over the past week the adolescents engaged in hard exercise that made them sweat and breathe heavily. PA ratings range from 0 to 7 days. SB was assessed by asking students how much time they usually spent watching TV or videos, playing video games, or playing on the computer both on school days and on Saturday and Sunday. The total number of hours spent in these sedentary activities was computed. | The adolescents rated their own health status in the past 12 months with the responses of ‘good, fairly good and not good’. | P+  Multiple linear regression.  For poor SRH, regression coefficient β (CI):  Boys: -0.39 (-0.57, -0.22); Girls: -0.31 (-0.50, -0.11). | NS  For poor SRH, β (CI):  Boys: 0.35 (-0.37, 1.08); Girls: 0.49 (–0.26, 1.24). | Sociodemographic factors, BMI, environmental factors, psychological factors (e.g., perceived stress, emotional symptoms). |
| Honkinen et al., 2005 Finland | Age range: 12 | Students were asked about the frequency of physical exercise and the degree of physical exercise for more than 30 min. Physical exercise was classified as adequate when it caused a person to sweat and breathe hardly for at least three times a week and for at least 30 min each time. Otherwise it was classified as inadequate. | School children were asked whether they perceived themselves as ‘very healthy, fairly healthy, or not very healthy’. For statistical analysis, the last two categories were combined. | P+  AOR (CI): For poor SRH, insufficient PA vs. sufficient PA: Total: 4.6 (2.66–7.89); Girls: 4.6 (1.93–10.77); Boys: 3.6 (1.77–7.16). | --- | Reported psychosomatic symptoms |
| Erginoz et al., 2004 Turkey | Age range: 15–20, Mean age = 16.4 (SD1.1) | PA was measured by hours a week that students exercise in free time to the extent that they get out of breath or sweat. Exercises were classified as two levels: 2–3 hours or more per week, less than 2 hours per week. | SRH was assessed by a single question “how healthy do you think you are?” Response choices: very healthy, quite healthy, not very healthy. The first two responses were combined into one group as ‘feeling healthy’ relative to ‘not very healthy’. | P+  AOR (CI), Exercise 2–3 h or more/wk (vs. <2 h/wk.): Girls: 1.869 (1.312–2.664); Boys: 1.533 (1.111–2.115). | --- | Daily smoking, headache, back ache, feeling dizzy, medication use, feeling left out of things, happiness, family well-off, parental support. |
| Pastor et al., 2003 Spain | Age range: 15–18, Mean age = 16.31 (SD0.92) | PA was measured by asking the subjects how often they participated in sports (excluding athletics at school), with scores ranged from 6 = 6–7 times a week to 1 = never. | SRH was measured on a four-point scale, ranging from 1 (not healthy at all) to 4 (very healthy). | P+  SEM regression. Sport participation: standardized coefficient B = 0.27, p<0.01. | --- | Mediators: tobacco and alcohol consumption, anxiety, depression, psychophysiologic symptoms. |
| Tremblay et al., 2003 Canada | Age range: 12–17 | PA was measured by frequency and duration of various sports activities during leisure time. The energy expenditure (EE) for each activity was calculated and summed to yield daily average EE Adolescents with leisure time EE below 1.5 were considered as inactive. | The question for SRH had five responses: excellent, very good, good, fair, poor. The variable was dichotomized to two levels: very good/excellent and good/fair/poor. | P+  AOR (CI) for good SRH: leisure time inactive vs. active: 12–14 years group: 0.75 (0.62–0.91); 15–17 years group: 0.65 (0.54–0.79). | --- | Sex, chronic conditions, obese, depression, household income, parental education, daily smoker, heavy drinking, fruit/vegetable, province. |
| Vingilis et al., 2002 Canada | Age range:12–19 | The energy expenditure was used to assess PA, and was divided into three groups based on the various activities lasting more than 15 minutes: active, moderate, and inactive, respectively. A binary variable was used comparing ‘active and moderate’ to ‘inactive’. | Single SRH question with five options: poor, fair, good, very good, excellent. A 4-point scale variable was used in the analysis by combining poor and fair into one category. | P+  AOR (CI) in log scale, PA inactive as reference group:  Moderate PA model 6: -0.276; Active PA model 6: -0.401. | --- | Ordinal logistic regression.  Age, sex, region, income, family structure, disability, social support, BMI, binge drinking, smoking, psychological distress. |
| Thorlindsson et al., 1990 Iceland | Age range: 15–16 | PA was measured by two variables. The subjects were asked how often they participated in sports each week (excluding athletics in school), and how many hours per week they spent participating in sports (excluding school athletics). The first variable ranges from 0 (no participation in sports) to 6 (six times/week or more), and the second variable ranges from 0 (no sport participation) to 5 (10 h or more in sports/week). | Perceived health status was assessed by a question with a four-point scale ranging from 1 (not healthy at all) to 4 (very healthy). | P+  Unstandardized β = 0.08, Standardized B = 0.195, p<0.001. | --- | SEM.  Mediators: smoking, alcohol, anxiety, depression, sychophysiological  symptoms. |
| **Longitudinal study** | |  |  |  |  |  |
| Burdette et al., 2017 US | 14-year follow up.  Age range: 14–18 at baseline, Mean age = 15.76 (SD1.27) | During the wave 1 (1995) in home interview, PA was measured as exercises at least three times in past week. Watching TV or playing video games no more than two hours per day in past week was considered not sedentary. Latent class analysis (LCA) was used to estimate health behaviour classes using the above variables with other health behaviours (sleep, diets, cigarettes smoking, drinking). Four health lifestyles classes were derived: low risk, moderate risk with substance use, moderate risk with inactivity, and high-risk. | SRH with five options: excellent, very good, good, fair and poor. Responses were coded such that a higher score indicates better perceived health. Both waves 1 (1995) and 4 (2008) data were used. | P+  For Wave 1 health: β = -0.41 (SE0.05), p<0.001 for the class group with high risk of physical inactivity (PIA). For Wave 4 health: (1) β = -0.24 (SE0.05), p<0.001 for the class group with high risk of PIA. (2) β = -0.15 (SE0.05), p<0.01 (further adjusted for wave 1 SRH. | --- | Multinomial Logistic regression.  Age, gender, race, residency, born place, region, parent education and health, household income, intact family structure, early childhood health challenges (early disability, low birth weight, parental alcoholism, smoker in household). |
| Liu et al., 2015 Japan | 6-year follow up.  Age range: 6 at Phase 2, Mean age = 12.25 (SD0.54) at Phase 4 | Frequency of participating in outdoor PA during a week, rated on 4 levels: very often, often, and occasionally, and never. PA was recoded as frequent (combining very often and often) and infrequent (combining occasionally and never). PA data of both in phases 2 and 4 were used. | In Phase 4 survey, participants were asked about their overall health over the past four weeks with 5 options: excellent, very good, good, fair, poor. The variable was dichotomized as: Good (excellent/very good/good) and Poor (fair/poor) health. | P+ for Good SRH vs. Poor.  Persisting physical active during the follow up (Yes vs. No).  UOR (CI): Total: 1.37 (1.17–1.60); Boys: 1.45 (1.14–1.85); Girls: 1.23 (1.00–1.51).  AOR (CI): Total: 1.27 (1.08 –1.50); Boys: 1.47 (1.14–1.89); Girls: 1.14 (0.92–1.42). | --- | Adjusted for frequency of eating breakfast and amount of sleep, headache and abdomen pain in the past 6 months. |
| Nigg et al. 2015 | 4-year follow up.  Mean age = 14.76 (SD 0.87) | MVPA was measured at both years (Y): Y1 and Y5. Children reported the number of days per week and the number of minutes per day they engaged in MVPA. The number of days in each level of PA was multiplied by the number of minutes each day spent in each level of PA. Total MVPA minutes per day were calculated based on the MVPA minutes per week. SB: children reported the number of hours per day they spent on TV viewing, playing video games, and using an internet (not for homework), with a response from 0 to 10 h. | General health question: “In general, I would say that my health is…”; five response levels from “1 = poor” to “5 = excellent.” | NS  Y1 MVPA did not significantly predict Y5 SRH. B = 0.01. | NS  SB at baseline (Y1) was not associated with SRH at Y5. B = -0.04. | Ethnicity |
| Spengler et al., 2014 Germany | 6-year follow up.  Age range: 11–17 (baseline) | Participants were asked about the amount and type of weekly PA in sports clubs and during leisure time. Sub-indices were calculated for every reported sport indicating the energy as metabolic equivalent of task (the METs) expended per week, and were added to an overall activity index. Media use was assessed as daily amount of time on watching TV, using a computer and playing console games. Food consumption was assessed with the food frequency questionnaire (FFQ) covering 54 food items. Four classes from the health-related behaviours (PA, media use and food consumption) were derived and characterized as: Cluster 1 (high PA level), Clusters 2 (high healthy nutrition score), Cluster 3 (very high media use), Cluster 4 (low scores on all included indices). | SRH had five response categories: very good, good, fair, poor and very poor. These answers were coded from 1 (very good) to 5 (very poor). Data on SRH at T1 (between 2003 and 2006) and T2 (2009 and 2012) were available for 953 participants aged 11 to 17 years at T1. | P+  The greatest improvement was observed in cluster 1  (high PA level).  Mean (SD) in SRH in cluster 1 (higher scores for SRH represents lower health level): T1: 1.78 (0.64); T2: 1.66 (0.65); change (T2–T1): -0.12 (t = 1.94, p = 0.055). | --- | NA (t-test) |
| Bauldry et al., 2012 US | 12-year follow up.  Age range: 11–19 at Wave 1, Mean age = 15.47 (SD1.69) | Asked the respondents how many days per week they engaged in various sports activities, including outdoor activities and organized sports as well as various forms of exercise. Respondents were classified physically inactive if they reported spending less than three days per week on any PA. | SRH was assessed at Wave 1 (year 1995), Wave 3 (2001–2002), and Wave 4 (2007–2009) by the question “In general, how is your health” with responses from excellent (5) to poor (1). | P+  Linear regression β = -0.129, SE = 0.010, p<0.001 (Not active vs. Active). | --- | Age, gender, race, education/diseases/alcoholism of parent, years of education, physical abuse, early disability, low birth weight, body weight, drink, depression, household smoker. |
| Elinder et al., 2011 Sweden | 3-year follow up.  Age range: 15, Mean age = 15.6 (SD0.3) | Students in grade 9 were asked about MVPA with the question: "How many hours per week in your leisure time do you move so intensively that you become out of breath or sweat?" Six options were from1⁄2 hour to 6 hours. PA was divided into three categories: >4 h/week, 2–4 h/week, and <2 h/week. Students in grade 12 were asked the question "Do you exercise regularly at least 1 hour per week?" with an answer of ‘yes’ or ‘no’. | Students in grade 12 were asked about their SRH "How healthy do you feel?" with three options: very healthy, quite healthy and not very healthy. The outcome was dichotomized to Good SRH (very healthy/quite healthy) and Poor SRH (not very healthy). | P+ for boys, NS for girls.  PA>4 h/wk as reference group.  UOR (CI): Boys: PA 2–4 h/wk: 16.92 (2.21–129.19), <2 h/wk: 15.41 (1.78–132.85). Girls: PA 2–4 h/wk: 1.07 (0.54–2.12), <2 h/wk: 1.98 (0.95–4.14).  AOR (CI): Boys: PA 2–4 h/wk: 16.04 (2.09–123.33), <2 h/wk: 14.69 (1.69–127.13). Girls: PA 2–4 h/wk: 0.99 (0.49–1.99), <2 h/wk: 1.56 (0.72–3.37). | --- | BMI in ninth grade, current smoking, and length of parents’ education. |
| JerdÊn et al., 2011 Sweden | 2-year follow up.  Age range: 12–13 | “Physical exercise” was measured by the question "How often do you usually exercise in your spare time (i.e. outside school) so you become breathless or sweating?", using a 7-grade ordinal scale. Other PA was measured by the question "How often do you do other things on your spare time (i.e., outside school) that increases your physical fitness, as walking, cycling, roller blades, dancing etc?", using a 7-grade ordinal scale. The answer of ‘two or three times weekly’ or ‘more often’ was classified as ‘high’, and the remaining answer as ‘low’ for both physical exercise and other PA. | SRH was measured by the question "How do you consider your well-being most of the time?", using a 5-grade ordinal scale: very good, rather good, nor good nor bad, rather bad, very bad. SRH was classified into three levels: ‘high’(very good), ‘medium’(rather good), and ‘low’ (nor good nor bad, rather bad, or very bad). | P+  Frequent physical exercise vs. low: UOR (CI):  Boys: 1.17 (1.02–1.35), Girls: 1.20 (1.03–1.39).  AOR (CI): Boys: 1.15 (0.96–1.37), Girls: 1.12 (0.92–1.37). | --- | Ordinal logistic regression.  Empowerment, self-esteem, school experience, mood in family, support from family, SRH in seventh grade. |
| Breidablik et al., 2009 Norway | 4-year follow up.  Age range: 13–19 | Outside school PA was evaluated by the following indicators: (1) how many hours a week do you play sport, or exercise to the point where you breathe heavily and/or sweat? (2) Are you actively involved in sports? | SRH question: “How is your overall health at the moment?”, with the answer categories: very good, good, not very good and poor. The ‘Poor’ was combined with ‘Not very good’ in the analyses. SRH data were available in both T1 (time 1: 1995–1997) and T2 (time 2: 2000–2001). | P+  Ordinal logistic regression.  AOR (CI), covariates at T1 and SRH at T2:  (1) adjusted for SRH at T1:  Lack of sports and exercise: 1.57 (1.43–1.72).  (2) adjusted for all covariates and SRH at T1):  Lack of sports and exercise: 1.64 (1.45–1.86). | --- | SRH at T1, gender, age, family divorce, health complaints, medical diagnosis, disability, health services, mental health, self-esteem, general well-being, body concern, school relationship, health-compromising habits. |
| Sacker et al., 2006 UK | 15- to 17-year follow up for 1970 and 1958 birth cohorts, respectively.  Age range:16 at baseline, 30 and 33 years at follow up | Leisure-time PA at 16 years. In the NCDS, PA was measured with three questions on participation in indoor sports, outdoor sports and swimming. In the BCS70, two questions on playing sports at clubs or centres and on the street or in a park or  playground are used. The summed score of the items was recoded with a range from 0 to 6; a higher score indicates higher frequency of PA. | Self-assessed overall health in adulthood was on a 4-point scale from excellent, good, fair or poor health. It was dichotomized into 0 for excellent/good health and 1 for fair/poor health. | P+ for NCDS (1958 birth cohort) only.  PA in adolescence for SRH in adulthood: AOR (CI): NCDS Men: 0.92 (0.88–0.97), Women: 0.93 (0.89–0.98); BCS70 Men: 0.97 (0.92–1.02), Women: 0.95 (0.90–1.00). UOR (CI): NCDS Men: 0.92 (0.87–0.97), Women:0.94 (0.89–0.99); BCS70 Men: 0.98 (0.93–1.03), Women 0.96 (0.91–1.02). | --- | Material disadvantage, BMI, psychosocial problems. |

PA-physical activity, MVPA-moderate to vigorous physical activity, SB-sedentary behavior, SED-sedentary, SRH-self-rated health, SE-standard error, SD-standard deviation, , PC-personal computers; TV-television; P+: positive association between PA and SRH, N-: negative association between SB and SRH, NS: not a statistically significant association, OR-odds ratio, CI-confidence interval (95% CI was reported), AOR-adjusted odds ratio, UOR-unadjusted odds ratio, BMI-body mass index, ---: Not applicable, Age measured as years; B-linear regression standardized coefficient; β-linear regression unstandardized coefficient; wk-week; h-hours; vs.-versus.
