# Supplementary material for: Associations between physical activity, sedentary behaviour and self-rated health among the general population of children and adolescents: a systematic review and meta-analysis
Source: BMC Public Health. 2020 Sep 3;20:1343. doi: 10.1186/s12889-020-09447-1 (PMC7650260; doi:10.1186/s12889-020-09447-1)
Supplement: Supplementary file 4 — Additional file 4. Fig. A–D funnel plots: comparisons of the odds of “poor” SRH by PA and SB. [file 12889_2020_9447_MOESM4_ESM.doc]

**Figures A-D funnel plots: comparisons of the odds of** "**poor**" **self-rated health (SRH) by physical activity (PA) and sedentary behaviour (SB) among children and adolescents**

| **Fig. A Funnel plot of PA and SRH for total sample** |
| --- |
|  |

OR: odds ratio, SE: standard error

| **Fig. B Funnel plot of PA and SRH by gender** |
| --- |
|  |
| 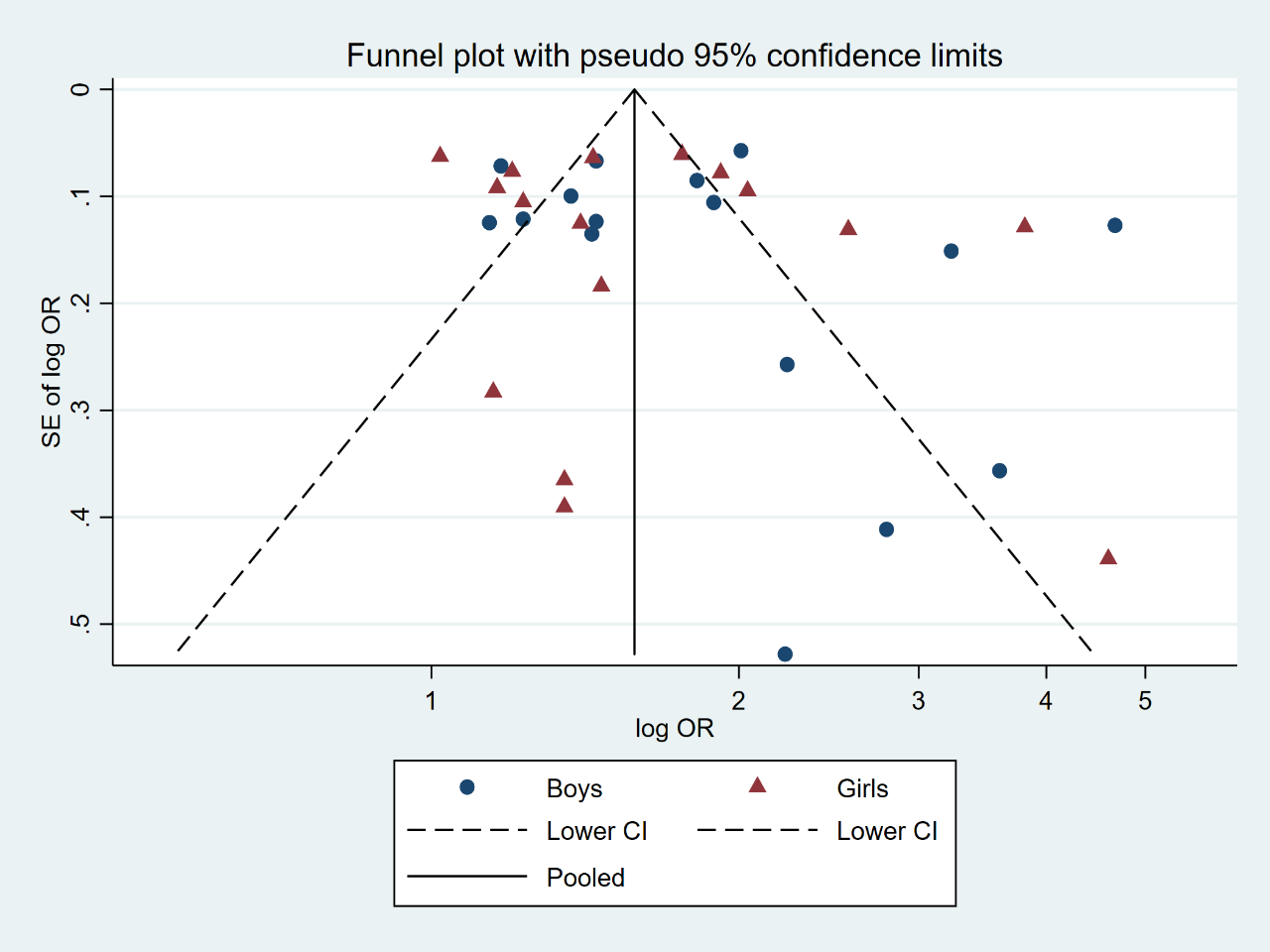 |

OR: odds ratio, SE: standard error

| **Fig. C Funnel plot of SB and SRH for total sample** |
| --- |
|  |

OR: odds ratio, SE: standard error

| **Fig. D Funnel plot of SB and SRH by gender** |
| --- |
|  |

OR: odds ratio, SE: standard error
